# Metabolic reprogramming of Kaposi’s sarcoma associated herpes virus infected B-cells in hypoxia

**DOI:** 10.1371/journal.ppat.1007062

**Published:** 2018-05-10

**Authors:** Rajnish Kumar Singh, Fengchao Lang, Yonggang Pei, Hem Chandra Jha, Erle S. Robertson

**Affiliations:** 1 Departments of Otorhinolaryngology-Head and Neck Surgery and Microbiology, Perelman School of Medicine, University of Pennsylvania, Philadelphia, United States of America; 2 Discipline of Biosciences and Biomedical Engineering, Indian Institute of Technology, Indore, Madhya Pradesh, India; Harvard University, UNITED STATES

## Abstract

Kaposi’s sarcoma associated herpesvirus (KSHV) infection stabilizes hypoxia inducible factors (HIFs). The interaction between KSHV encoded factors and HIFs plays a critical role in KSHV latency, reactivation and associated disease phenotypes. Besides modulation of large-scale signaling, KSHV infection also reprograms the metabolic activity of infected cells. However, the mechanism and cellular pathways modulated during these changes are poorly understood. We performed comparative RNA sequencing analysis on cells with stabilized hypoxia inducible factor 1 alpha (HIF1α) of KSHV negative or positive background to identify changes in global and metabolic gene expression. Our results show that hypoxia induces glucose dependency of KSHV positive cells with high glucose uptake and high lactate release. We identified the KSHV-encoded vGPCR, as a novel target of HIF1α and one of the main viral antigens of this metabolic reprogramming. Bioinformatics analysis of vGPCR promoter identified 9 distinct hypoxia responsive elements which were activated by HIF1α *in-vitro*. Expression of vGPCR alone was sufficient for induction of changes in the metabolic phenotype similar to those induced by KSHV under hypoxic conditions. Silencing of HIF1α rescued the hypoxia associated phenotype of KSHV positive cells. Analysis of the host transcriptome identified several common targets of hypoxia as well as KSHV encoded factors and other synergistically activated genes belonging to cellular pathways. These include those involved in carbohydrate, lipid and amino acids metabolism. Further DNA methyltranferases, DNMT3A and DNMT3B were found to be regulated by either KSHV, hypoxia, or both synergistically at the transcript and protein levels. This study showed distinct and common, as well as synergistic effects of HIF1α and KSHV-encoded proteins on metabolic reprogramming of KSHV-infected cells in the hypoxia.

## Author summary

Hypoxia inducible factors (HIFs) play a critical role in survival and growth of cancerous cells, in addition to modulating cellular metabolism. Kaposi’s sarcoma associated herpesvirus (KSHV) infection stabilizes HIFs. Several factors encoded by KSHV are known to interact with up or downstream targets of HIFs. However, the process by which KSHV infection leads to stabilized HIF1α and modulation of the cellular metabolism is not understood. Comparative RNA sequencing analysis on cells with stabilized hypoxia inducible factor 1 alpha (HIF1α), of KSHV negative or positive cells led to identification of changes in global and metabolic gene expression. Our results show that hypoxia induces glucose dependency of KSHV positive cells with high glucose uptake and high lactate release. KSHV-encoded vGPCR was identified as a novel target of HIF1α regulation and a major viral antigen involved in metabolic reprogramming. Silencing of HIF1α rescued the hypoxia associated phenotype of KSHV positive cells. Analysis of the host transcriptome identified several common targets of hypoxia and KSHV-encoded factors, as well as other synergistically activated genes belonging to cellular metabolic pathways. This study showed unique, common and the synergistic effects of both HIF1α and KSHV-encoded proteins on metabolic reprogramming of KSHV-infected cells in hypoxia.

## Introduction

Kaposi’s sarcoma associated herpesvirus (KSHV) is the etiological agent of Kaposi sarcoma, primary effusion lymphoma and multicentric Castleman disease [1–3]. By altering the expression of core metabolic enzymes, KSHV infected cells acquire a metabolic strategy of aerobic glycolysis generally referred as to the Warburg effect where these cells drive a high rate of glycolysis even in the presence of molecular oxygen [4–8]. This alteration of host metabolism mimicking Warburg effect by KSHV is believed to be necessary for the maintenance of latently infected cells [4]. Similar to most cancer cells, the mitochondria is also an organelle targeted by KSHV in viral infected cells altering apoptotic pathways and metabolism, so necessitating up-regulation of glycolysis to compensate for the energy demands of rapidly growing cells [9–12].

Metabolite profiling of KSHV infected cells suggest a wide difference between metabolite pools of KSHV infected cells when compared to control cells, including those which are common to anabolic pathways of most cancer cells [5]. Interestingly, the metabolite changes are not limited to only carbohydrates, but also included fatty acids and amino acids where inhibition of key enzymes in this pathway led to apoptosis of infected cells [5,13]. KSHV infection-mediated elevation of metabolites pools are due to enhanced anabolic activity rather than degradation from respective macromolecules [5]. Previous attempts to identify the mechanism of such reprogramming confirm increased expression of host factors such as glucose transporters, as well as hypoxia inducible factor (HIF1α) which are prerequisites for such changes in KSHV infected cells. In addition, decreased mitochondrial copy number and down regulated EGLN2 and HSPA9 have been reported upon over-expression of KSHV coded microRNAs, and are believed to be among the many KSHV factors involved in metabolic changes [14]. Nevertheless, previous observations either do not support or was unable to determine the involvement of other KSHV-encoded factors involved in metabolic differences caused by KSHV infection.

Hypoxia and HIF1α play critical roles in pathogenesis of KSHV by modulating expression of KSHV genes as well as stabilizing several KSHV-encoded proteins [15,16]. KSHV infection alone can mimic several physiological and metabolic changes due to hypoxia and those common to cancer cells. Hypoxia on the other hand plays an important role in KSHV reactivation biology where HIF1α facilitates KSHV-encoded RTA-mediated reactivation by binding with LANA and up-regulating RTA expression [16,17]. Hypoxia is also reported to enhance viral reactivation potential associated with 12-O-tetradecanoylphorbol-13-acetate [18]. The role of hypoxia in maintenance of latency and KSHV associated pathogenesis is also crucial, where the promoter of the key latent gene cluster coding for LANA, vFLIP and vCyclin harbors hypoxia responsive elements and can be activated by HIF1α [15]. Among other KSHV factors affecting the HIF1α axis, is the constitutively active G protein-coupled receptor (vGPCR) encoded by KSHV [19,20]. vGPCR is a bonafide oncogenic protein and stimulates angiogenesis by increasing the secretion of vascular endothelial growth factor (VEGF), which is a key angiogenic stimulator and a critical mitogen for the development of Kaposi’s sarcoma [21,22]. KSHV-encoded vGPCR enhances the expression of VEGF by stimulating the activity of the transcription factor HIF1α, which activates transcription from a HRE within the 5'-flanking region of the VEGF promoter [23]. Stimulation of HIF1α by KSHV encoded vGPCR involves phosphorylation of its regulatory/inhibitory domain by p38 and mitogen-activated protein kinase (MAPK) signaling pathways, thereby enhancing its transcriptional activity [24]. Specific inhibitors of the p38 / MAPK pathways are able to inhibit the transactivating activity of HIF1α induced by the KSHV-encoded vGPCR, as well as the VEGF expression and secretion from cells expressing this receptor [24]. These findings suggest that the KSHV-encoded vGPCR oncogene subverts convergent physiological pathways leading to angiogenesis and provides the first insight into a mechanism whereby growth factors and oncogenes acting upstream of MAPK, as well as inflammatory cytokines and cellular stresses that activate p38, can interact with the hypoxia-dependent machinery of angiogenesis [24]. However, the role of vGPCR in modulating other physiological pathways is poorly explored.

In the present study, we investigated the role of stabilized HIF1α on the metabolic status of KSHV positive cells and compared the results with KSHV negative cells with the same genetic background under normoxic or hypoxic conditions. We present data for differentially expressed KSHV-encoded genes when HIF1α is stabilized. We then showed the changes in global transcription of cells growing in normoxia or hypoxia with HIF1α to identify the common targets of HIF1α and KSHV infection. Our results showed enhanced induction of a tumorigenic metabolic phenotype in KSHV-positive cells growing in hypoxia compared to KSHV-negative cells growing under the same condition. Further, we now identify a comprehensive list of metabolic genes differentially expressed on KSHV infection in the hypoxic environment. These results provide new insights into the role of KSHV factors, in cooperation with hypoxia on the global metabolic status of KSHV positive cells.

## Results

### KSHV infection induces a pro-cancerous phenotype in hypoxia

**Fig 1:**
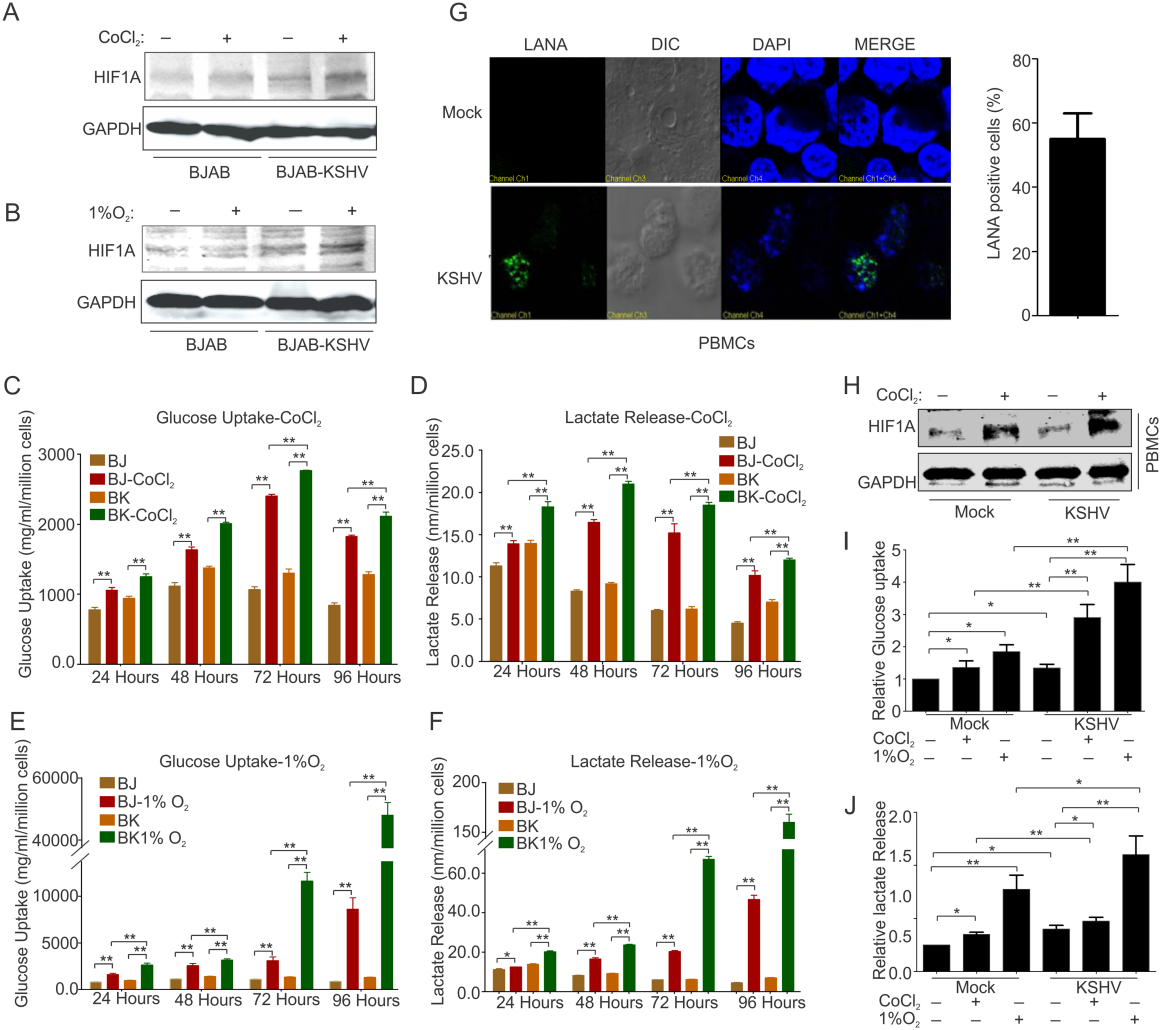
Metabolic characterization of KSHV negative/positive cells growing in normoxia and CoCl_2_ or 1% O_2_ induced hypoxia. (A&B) Western blot analysis of HIF1α for the confirmation of hypoxia induction in BJAB and BJAB-KSHV cells growing in the presence of CoCl_2_ or1% O_2_. (C) Time dependent glucose uptake estimation in culture media from BJAB/BJAB-KSHV cells growing in normoxia or CoCl_2_ induced hypoxia. (D) Time dependent lactate released estimation in culture media of BJAB/BJAB-KSHV cells growing in normoxia or CoCl_2_ induced hypoxia. (E) Time dependent glucose uptake estimation in culture media from BJAB/BJAB-KSHV cells growing in normoxia or 1% O_2_ induced hypoxia. (F) Time dependent lactate released estimation in culture media of BJAB/BJAB-KSHV cells growing in normoxia or 1% O_2_ induced hypoxia. (C), (D), (E) and (F) represent mean of three independent experiments. Asterisk (*) indicates differences which are statistically significant (BJ = BJAB, BK = BJAB-KSHV, * p≤0.05, **≤0.01). (G). LANA immune-staining showing KSHV infection of PBMC. The bar diagram represents KSHV infected cells as measured microscopically for LANA immune-staining. (H) Western blot analysis of HIF1α for the confirmation of hypoxia induction in control and KSHV infected PBMCs growing in the presence of CoCl_2_. (I) Glucose uptake estimation in control and KSHV infected PBMCs at 48 hours post infection and grown under normoxia or CoCl_2_ /1% O_2_ induced hypoxia. (J) Estimation of lactate released by control and KSHV infected PBMCs at 48 hours post infection and grown under normoxia or CoCl_2_ /1% O_2_ induced hypoxia. The mean values from individual experiments were used to plot graph and the error bars were calculated based on differences from mean value (* p≤0.05, **≤0.01).

KSHV infection is known to stabilize HIFs and this stabilization provides the cells a mechanism to survive in a hypoxic environment by up-regulating several cellular pathways involved in metabolism, survival and angiogenesis. We wanted to determine how KSHV infected cells respond compared to their isogenic KSHV-negative counterparts in hypoxic environments, and their metabolic requirements. There is no available control cell line with the same isogenic background for comparative studies in B-cells. Therefore, we selected KSHV-negative BJAB cells and KSHV positive BJAB-KSHV stably infected with KSHV [25]. We first characterized and confirmed the presence of full length KSHV in BJAB-KSHV cells at the level of the viral genome and transcriptome to determine if gross genomic alterations had occurred. Amplification of 10 different KSHV genomic regions with KSHV specific primers confirmed the presence of a KSHV genome most likely intact in BJAB-KSHV cells (S1A and S1B Fig). The sequences of the primers used to characterize BJAB-KSHV cells are also provided in S1 Table. The BJAB-KSHV cells were further characterized at the level of transcripts by amplifying the KSHV-encoded latent gene vCyclin from the cDNA made from BJAB-KSHV cells (S1C Fig). The isogenic background and authenticity of these two cell lines were also examined by short tandem repeat (STR) profiling. The STR profiling results for these two cell lines were compared with each other as well as with BJAB cells STR profile obtained from ExPASy Bioinformatics Resource portal database. The STR profile results confirmed the same origin and isogenic background of these cells (S2 Table). To study the effects of hypoxia, we proceeded with two different approaches to induce hypoxia in cell culture. In the first approach, we treated the cells with CoCl_2_, a chemical inducer of hypoxia to induce stabilization of HIF1α with minimal effects on the growth rate of the cells [26]. In the second approach we grew cells in 1% O_2_ hypoxic condition. Puromycin was omitted from the media of BJAB-KSHV and control cells for the entire treatment period. HIF1α stabilization was confirmed by western blot using HIF1α specific antibody (Fig 1A and 1B). An estimation of glucose consumed by BJAB or BJAB-KSHV cells suggested that the bulk of glucose from medium was being consumed during the initial period of 24–48 hours, in which cells growing under normoxic conditions showed an exponential growth pattern (Fig 1C & 1E). The growth patterns of cells growing in either CoCl_2_ or 1% O_2_ were quite different and showed diminished proliferation rates. Growing the cells in the same partially depleted medium showed a retarded growth in both normoxic as well as hypoxic environments, though hypoxic induction due to low oxygen showed a more drastic adverse effects on cell survival (Fig 1C & 1E). The estimation of glucose consumed by BJAB and BJAB-KSHV cells grown in normoxia and hypoxia showed a large difference in the consumption of glucose between these cells. Within the initial 24 hours, BJAB-KSHV cells showed an almost 18% higher glucose consumption as compared to BJAB cells during the same time period (940.7mg compared to 773.8mg glucose per million cells) (Fig 1C & 1E). The BJAB-KSHV cells showed a similar increase in uptake of glucose throughout the time points of 48, 72 and 96 hours compared to BJAB cells. A time dependent enhancement in the glucose uptake was observed for BJAB-KSHV cells when compared to BJAB cells growing in normoxic condition. However, the diminished medium condition and hypoxia due to 1% oxygen led to a drastic reduction in cell survival and growth past 72 hours. To rule out the possibility of an effect of puromycin pretreatment on glucose uptake, glucose consumption was also measured in cells growing either in the presence of puromycin, or its absence for 48 hours. The results showed no significant difference in glucose consumption due to presence or absence of puromycin in culture of BJAB-KSHV cells (S1D Fig). The effect of puromycin due to hypoxic induction or its downstream target was also determined by measuring real time expression of HIF1α and VEGFA. The results showed no effects of puromycin on expression of HIF1α nor VEGFA (S1E Fig).

To estimate lactate released in medium by these cells a standard curve of lactate ranging from 0 to 10 nmol/μl was prepared followed by a pilot experiment to determine the range of lactate in the medium. Here, different volumes of fresh culture medium (1μl and 10 μl) and 1μl medium from growing cultures were used (S1F Fig). Based on the pilot experiment, 10 μl of a 10X diluted culture medium was used to estimate lactate released in medium by BJAB and BJAB-KSHV cells growing under normoxia or CoCl_2_/1%O_2_-induced hypoxia (Fig 1D and 1F). A pattern similar to glucose uptake was observed for lactate release in these cells under similar growth conditions suggesting a directly proportional relationship between glucose uptake and lactate release. We also investigated whether this metabolic phenotype was mimicked in primary infection to peripheral blood mononuclear cells, KSHV infection of PBMCs was monitored growing them in the presence of CoCl_2_ or 1%O_2_. The infection of PBMCs with KSHV was confirmed by immuno-staining for KSHV latent protein LANA and the induction of hypoxia was confirmed by western blot to detect HIF1α (Fig 1G and 1H). The percentage of cells infected with KSHV was empirically calculated by LANA immune-staining. The infection efficiency of PBMCs with KSHV was approximately 50%. Estimation of glucose uptake and lactate release by infected PBMCs grown under conditions of normoxia or in CoCl_2_ or 1%O_2_ at 48 hours post-infection showed an enhanced glucose dependency and lactate release similar to BJAB and BJAB-KSHV cells (Fig 1I and 1J).

### Differential expression of KSHV encoded genes in hypoxia

**Fig 2:**
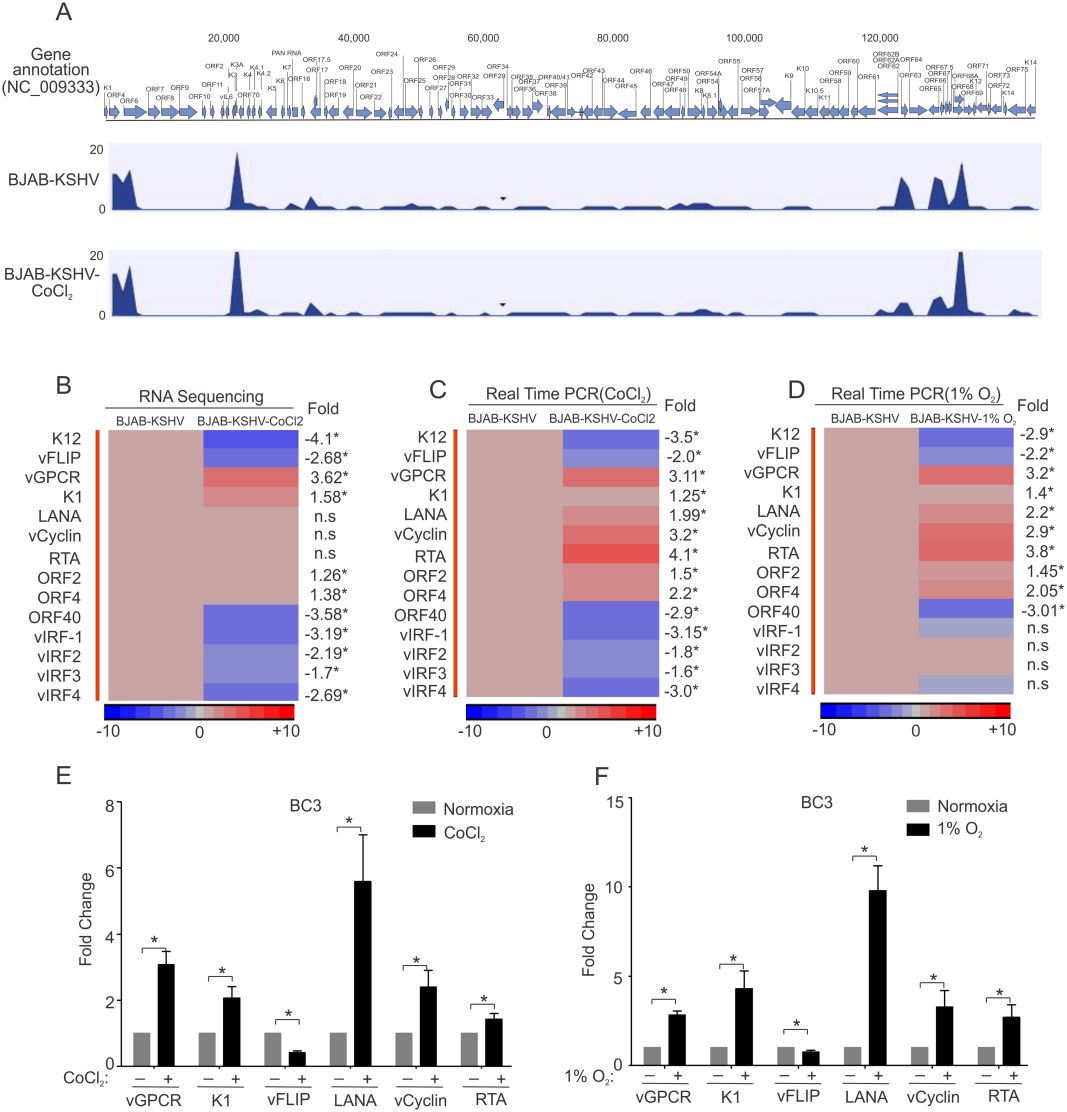
Differential expression of KSHV encoded gene expression in CoCl_2_/1%O_2_ induced hypoxia. BJAB-KSHV cells were grown under normoxic or CoCl_2_ induced hypoxic environment for 48 hours followed by RNA isolation. The duplicate RNA samples were prepared for sequencing using Illumina RNA sequencing sample prep kits. The indexed ready to run samples were run on Illumina platform at the University of Washington (Core services). The reads alignment with KSHV genome, fold change differences and statistical analysis in terms of p-value were calculated by CLC bio software (Qiagen Inc., Hilden, Germany). The differences in fold change were represented by heat maps using Partek software. (A) Histogram showing transcript coverage for KSHV encoded genes. (B) Heat map for fold change expression of KSHV-encoded genes based on analysis of RNA sequencing data. Asterisk (*) denotes statistical significance with p<0.05; (n.s denotes non-significant difference). (C) Validation of RNA sequencing data by real time PCR in CoCl_2_ induced hypoxia. BJAB-KSHV cells were grown under normoxic or CoCl_2_ induced hypoxia followed by RNA isolation and real time PCR. Histogram represents real time PCR fold change of reverse transcribed KSHV encoded genes transcript to cDNA of BJAB-KSHV cells grown in CoCl_2_ induced hypoxia. Asterisk (*) denotes statistical significance with p<0.05. (D) Validation of RNA sequencing data by real time PCR in 1% O_2_ induced hypoxia. BJAB-KSHV cells were grown under normoxic or 1% O_2_ induced hypoxia followed by RNA isolation, conversion to cDNA and real time PCR. Histogram represents real time PCR fold change of KSHV encoded transcripts converted to cDNA in BJAB-KSHV cells growing in 1% O_2_ induced hypoxia. Asterisk (*) denotes statistical significance with p<0.05; (n.s denotes non-significant difference). (E & F) Fold change expression of KSHV encoded vGPCR, K1, vFLIP, LANA, vCyclin and RTA in KSHV positive BC3 cells grown under normoxia and CoCl_2_/1% O_2_ induced hypoxia. BC3 cells were grown under normoxia or CoCl_2_ induced hypoxia (48 hours) or 1% O_2_ (24 hours) followed by RNA isolation and cDNA synthesis. Real-time PCR assays were performed using gene specific primers. All the experiments were performed at least in triplicate and the statistical significance in the terms of p-value were determined using graph pad software. Asterisk (*) denotes statistical significance with p<0.05.

As the 1% oxygen for induction of hypoxia showed highly adverse effects on cell survival, we performed RNA sequencing experiments on BJAB and BJAB-KSHV cells growing in normoxia or CoCl_2_-induced hypoxia showing a more relevant physiological response of HIF1α stabilization due to KSHV infection. Analysis of RNA sequencing data for differential gene expression of KSHV encoded genes identified 42 transcripts coded by KSHV (Fig 2A–2D). A histogram for genes across the KSHV genome is provided in Fig 2A. Statistical analysis revealed that expressions of 11 KSHV-encoded genes were significantly changed when grown under CoCl_2_–induced hypoxia compared to their normoxic counterpart. Among these 11 genes, the viral G-protein coupled receptor (vGPCR), which is a constitutively active homolog of human G-protein coupled receptor [24], was found to be up-regulated by 3.62 fold (Fig 2B). Three other genes up-regulated due to hypoxia were K1 (Immunoreceptor tyrosine-based activation motif containing signal transducing membrane protein), ORF2 (homolog of cellular Dihydrofolate reductase), and ORF4 (Complement binding protein) with a fold change of 1.58, 1.26 and 1.38, respectively (Fig 2B–2D). Among the down-regulated genes, K12, ORF 40, and vFLIP were heavily down-regulated with a fold change of -4.1, -3.58 and -2.68, respectively (Fig 2B–2D). The levels of LANA and vCyclin transcripts were induced but not statistically significant due to possible differential efficiency of sequencing through these templates (Fig 2B). However, they were clearly induced as shown by RT-PCR of cells grown in CoCl_2_ and 1% O_2_ (Fig 2B & 2C). RTA transcripts were moderately increased as detected by sequencing, but was clearly increased when validated by RT-PCR in CoCl_2_ and 1% O_2_ (Fig 2B & 2C). Interestingly, all the four KSHV encoding interferon regulatory factors (vIRFs) were down-regulated with a fold change of -3.19, -2.19, -1.7 and -2.69 for vIRF1, vIRF2, vIRF3 and vIRF4, respectively (Fig 2B–2D). To validate the results obtained from differential gene expression seen for KSHV-encoded genes by RNA-sequencing, real-time PCR was also performed for the individual genes using gene specific primers. The primers used for real-time PCR are provided in S3 Table. Similar results were obtained by real-time PCR assays where vGPCR showed the highest up-regulation and K12 as a greatest down-regulated gene (Fig 2B & 2C). Similarly, RTA was shown to be up-regulated by RT-PCR in CoCl_2_ and 1% O_2_, as expected (Fig 2B & 2C). To further corroborate the differential gene expression of KSHV-encoded genes, we wanted to determine if a similar pattern was observed in low oxygen environment. BJAB-KSHV cells grown in a hypoxic chamber with 1% oxygen were collected and real-time PCR analysis was performed on the KSHV-encoded genes. The results showed a similar pattern of expression for the genes analyzed. However, the magnitude of change was slightly lower for vGPCR while it was slightly higher for K1 as compared to their expression in CoCl_2_-induced hypoxia (Fig 2C & 2D). The expression of ORF2, ORF4, vFLIP, vCyclin, LANA and RTA was also observed with the same pattern as it was seen in CoCl_2_-induced hypoxia (Fig 2C & 2D). Interestingly, the expression of some of the vIRFs were slightly less than that observed in CoCl_2_-induced hypoxia suggesting that the expression of vIRFs are also dependent on the overall ATP pool (Fig 2D). To determine the physiological relevance of the differentially expressed KSHV-encoded genes in response to hypoxia, real time expression of vGPCR, K1, vFLIP, vCyclin, LANA and RTA were also analyzed in the primary effusion lymphoma (PEL) cell line BC3, grown in both CoCl_2_ as well as 1% O_2_ induced hypoxia. The results strongly supported a universal effect of hypoxia on the expression of these KSHV-encoded genes (Fig 2E & 2F). These results led to further analysis of other critical KSHV-encoded genes when HIF1α was stabilized in the naturally infected KSHV positive cell line, BC3. We analyzed expression of 27 candidate KSHV-genes from BC3 cells grown under normoxic and CoCl_2_ induced hypoxic condition. The primer sets used are included in S3 Table. The resulting data showed that ORF9 (DNA polymerase), ORF18 (involved in late gene regulation), ORF25 and ORF26 (major capsid protein), ORF27 (Glycoprotein), ORF28 (BDLF3 EBV homolog), ORF34, ORF40 (Helicase-primase), ORF57 (mRNA export/splicing), and ORFK14.1 were significantly up-regulated in BC3 cells grown under hypoxic conditions (S2A–S2C Fig). Similarly, ORF11 (predicted dUTPase), ORF31 (nuclear ad cytoplasmic protein), ORF32, ORF33 (tegument proteins), ORF44 (Helicase), ORF64 (Deubiquitinase) and ORFK14 (vOX2) were significantly down-regulated in BC3 cells grown under hypoxic condition (S2A–S2C Fig). The expression of ORF6 (ssDNA binding protein), ORF7 (virion protein), ORF8 (Glycoprotein B), ORF36 (serine protein kinase), ORF54 (dUTPase/Immunmodulator), ORF56 (involve in DNA replication), ORF69 (BRLF2 nuclear egress) and ORFK8.1 (Glycoprotein) showed little or no significant change (S2A–S2C Fig).

### Role of the HIF1α-vGPCR axis in metabolic changes associated with KSHV-infected cells

**Fig 3:**
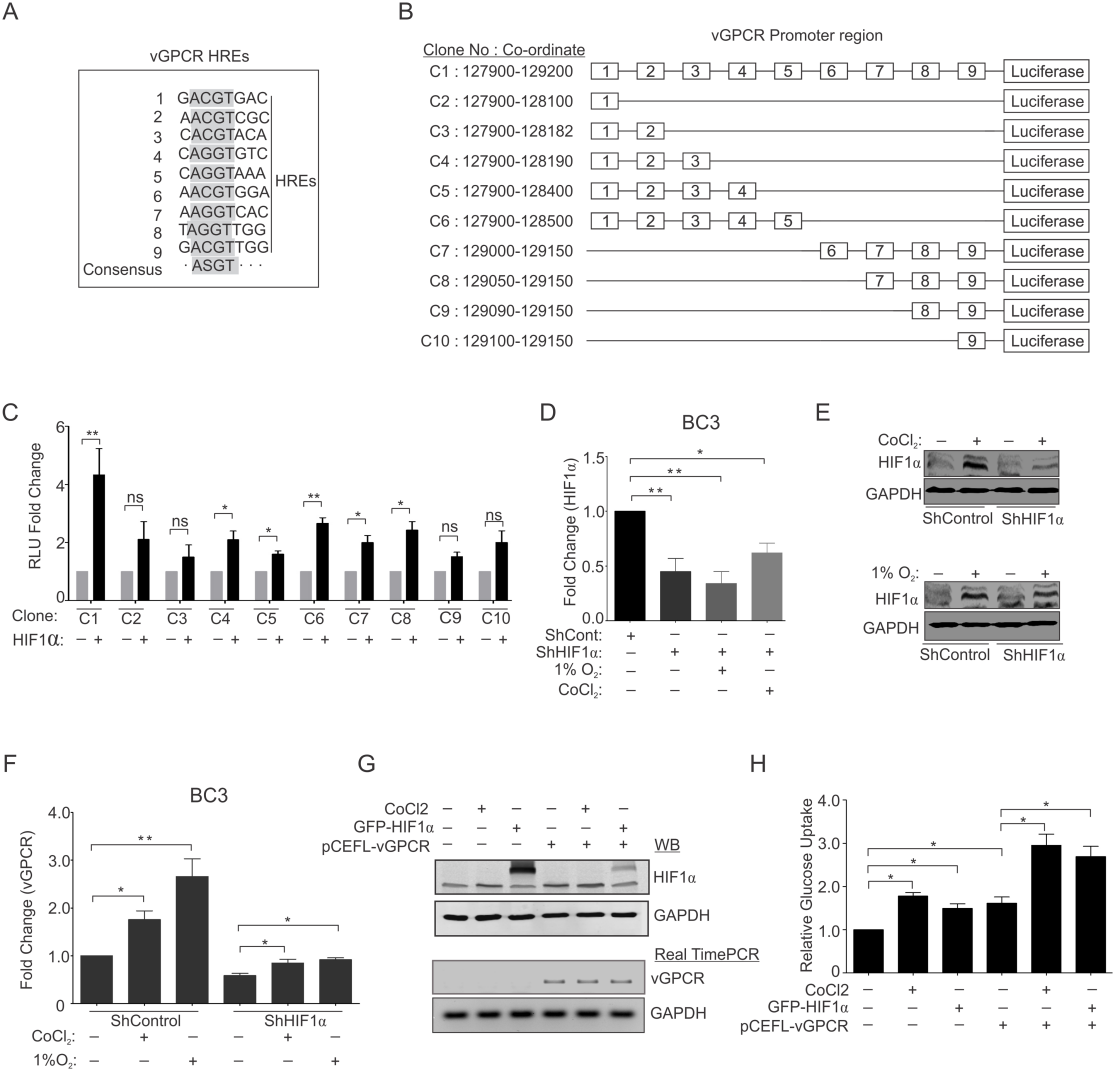
Role of HIF1α-vGPCR axis in metabolic changes of KSHV infected cells. (A) Representation of various HREs in the promoter region of vGPCR. vGPCR promoter region was scanned for presence of HREs with the consensus sequence (–ASGT---; where S = C/G;[16]). (B) Schematic representation of various clones of vGPCR promoter used for luciferase based reporter assay. (C) Relative luminescence unit of various promoters constructs in the absence/presence of over-expressed HIF1α. HEK293T cells were transfected with either promoter clone alone or co-transfected with HIF1α plasmid. The cells were lysed in passive lysis buffer and equal amounts of proteins were used for luciferase based assays. (D) Fold change expression of HIF1α in ShControl and ShHIF1α BC3 cells grown under normoxia or CoCl_2_/1%O_2_ induced hypoxia. BC3-ShControl and BC3-ShHIF1α were generated by lentiviral based transduction and selected under 2μg/ml puromycin. Cells were grown under normoxia or CoCl_2_/1%O_2_ induced hypoxia followed by RNA isolation, cDNA synthesis and HIF1α real time PCR. (E) Western Blot analysis for HIF1α in ShControl and ShHIF1α BC3 cells grown under normoxia or CoCl_2_/1%O_2_ induced hypoxia. Equal amount of whole cell lysates were used to probe for HIF1α. (F) Relative fold change expression of vGPCR transcripts in ShControl and ShHIF1α BC3 cells grown under normoxia or CoCl_2_/1%O_2_ induced hypoxia. (G) Western blot analysis of HIF1α and Real time PCR for vGPCR transcripts. (H) Relative glucose uptake in HIF1α/vGPCR over-expressed cells. HEK293T cells were transfected with HIF1α or vGPCR plasmids alone or in combination and the results of glucose uptake were compared with control cells or cells treated with CoCl_2_. The mean values from individual experiments were used to plot graph and the error bars were calculated based on differences from mean value (* p≤0.05, **≤0.01).

Based on the results showing HIF1α stabilization and up-regulated expression of vGPCR, we performed a bioinformatics analysis of the vGPCR promoter region for identification of possible hypoxia responsive elements (HREs) [16]. A search for HREs consensus (ASGT; where S = C/G) within the vGPCR promoter identified 9 different HREs (Fig 3A). To determine the role of these HREs in directly regulating transcription of vGPCR in a HIF1α dependent manner, luciferase based reporter assays were performed. In brief, 10 different clones from the promoter region of vGPCR were generated and the results from the luciferase activity showed that the HREs at the 3^rd^, 4^th^, 5^th^, 6^th^, and 7^th^ positions were significantly responsive to HIF1α (although not equally responsive). The promoter region containing all 9 HREs showed the strongest response, followed by clone C6 containing the initial 5 HREs (Fig 3C). The primers used to generate the clones are provided in S4 Table. Next, we wanted to determine if HIF1α knockdown in KSHV-positive cells can rescue the hypoxia associated expression of KSHV-encoded genes. The ShControl and ShHIF1α-BC3 cells were generated by lentivirus based transduction. Knock-down of HIF1α transcripts was confirmed at the transcript levels by real time PCR (Fig 3D). We confirmed the expression of HIF1α at the protein level by HIF1α western blot of lysates from ShControl and ShHIF1α BC3 cells grown under CoCl_2_ or 1% O_2_ induced hypoxia (Fig 3E). Real-time PCR analysis to determine the vGPCR and vFLIP expression in CoCl_2_ treated cells. HIF1α knockdown cells showed a reversal of expression as treatment with CoCl_2_ did not show the effect in HIF1α competent cells (Fig 3F and S2D Fig). As vGPCR is a potent candidate for the activation of several proliferation pathways, we wanted to determine whether the metabolic phenotype observed for KSHV positive cells was only due to elevated HIF1α levels, or if vGPCR expression was sufficient to induce the metabolic changes. We transfected HEK293T cells with an expression plasmid coding for KSHV-encoded vGPCR and compared it to that of cells transfected with empty vector. We also compared the results with cells expressing HIF1α. The results suggest that hypoxia or vGPCR can modulate the metabolic phenotype (Fig 3G and 3H).

### Transcriptional regulation due to hypoxia and KSHV-infection in B-cells

**Fig 4:**
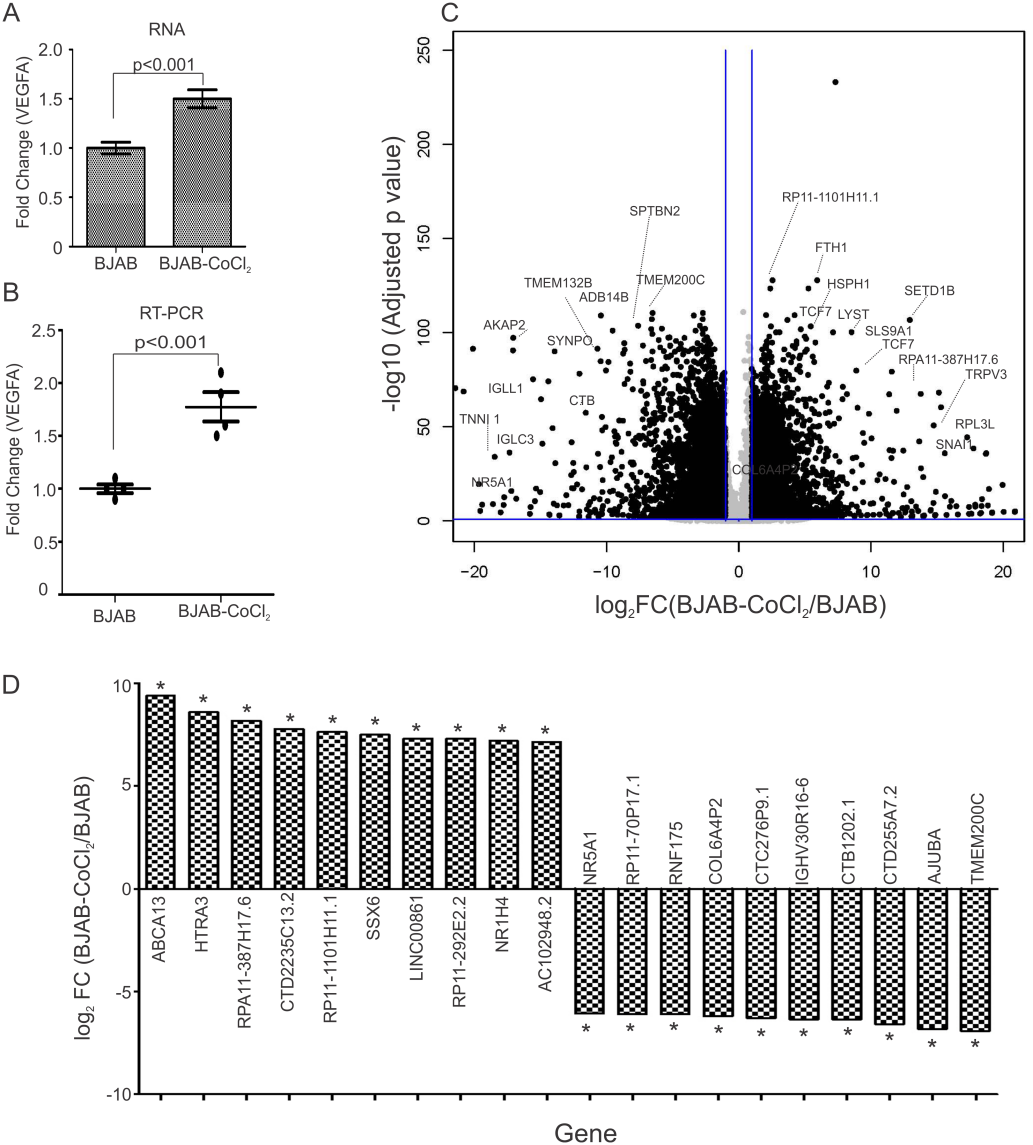
Differential gene expression between BJAB-CoCl_2_ and BJAB. The differences in expression of host encoded genes in terms of fold change between BJAB-CoCl_2_ and BJAB cells were determined by CLC bio software. BJAB cells were grown under normoxic or CoCl_2_ induced hypoxic condition for 48 hours followed by RNA isolation through standard phenol chloroform extraction. The RNA samples were prepared for sequencing using Illumina RNA sequencing sample prep kit as described in materials and methods section. The indexed ready to run samples were run on Illumina platform at the University of Washington (Core services). The reads alignments with human genome, fold change differences and statistical significance in the terms of p-value were calculated by CLC bio software (Qiagen Inc., Hilden, Germany). (A) Data was analyzed for Fold change expression of VGEFA, a positively regulated target of HIF1α. Graph represents fold change expression of VGEFA in BJAB-CoCl_2_ cells compared to BJAB cells (RNA sequencing). (B) Difference in VEGFA gene expression was confirmed by real time PCR in RNA sample used for sequencing. Graph represents fold change expression of VGEFA in BJAB-CoCl_2_ cells compared to BJAB cells (Real Time PCR data). (C) Volcano plot for differential gene expression between BJAB-CoCl_2_/BJAB. Fold change differences and FDR-p value for each gene were feeded to R-software to generate volcano plot. (D) Top 10 up-regulated genes and top 10 down-regulated genes in BJAB-CoCl_2_ cells compared to BJAB cells. Asterisk (*) denotes FDR-p-value <0.05.

RNA sequencing on total RNA from BJAB and BJAB-KSHV cells grown under normoxic condition or CoCl_2_ induced hypoxic condition was analyzed to determine the differential gene expression profiles. To increase our confidence in the differential gene expression data for host genes, the fold-change difference for VEGFA (a known target of HIF1α) was first analyzed (Fig 4A). Real time PCR validation was also performed for VEGFA transcripts from CoCl_2_ treated cells (Fig 4B). A comparative analysis was performed between BJAB vs BJAB-CoCl_2_, BJAB vs BJAB-KSHV and BJAB vs BJAB-KSHV-CoCl_2_ cells. The comparative analysis between BJAB cells grown under normoxia and CoCl_2_ induced hypoxia revealed major transcriptional changes between the two conditions (Fig 4C). CoCl_2_ treatment resulted in up-regulation of 2,182 transcripts (p≤0.001; FC ≤2 or ≥ 2). Similarly 1882 transcripts were observed down-regulated due to CoCl_2_ treatment (p≤0.001; FC ≤2 or ≥ 2). A volcano plot for the differentially expressed genes in BJAB-CoCl_2_ cells compared to BJAB cells is also presented showing that expression of a large number of genes was clearly modulated (Fig 4C).

**Fig 5:**
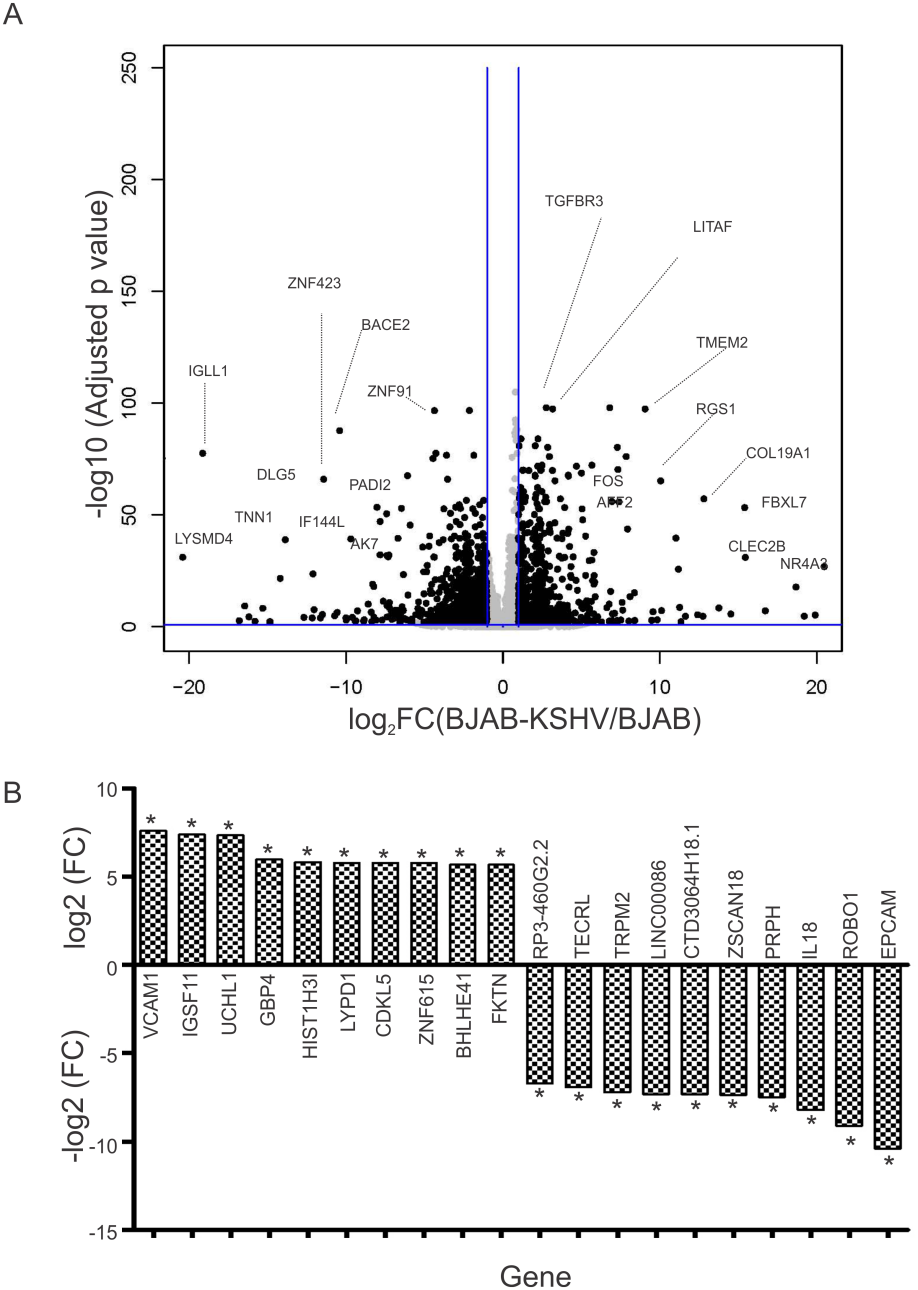
Differential gene expression between BJAB-KSHV and BJAB cells. RNA isolation, sample preparation and RNA sequencing were performed as described in materials and methods. The differences in expression of host encoded genes in terms of fold change between BJAB-KSHV and BJAB cells were determined by CLC bio software. (A) Volcano plot for differential gene expression between BJAB-KSHV and BJAB cells. Fold change differences and FDR-p value for each gene were fed into R-software to generate the volcano plot. (B) Top 10 up-regulated genes and top 10 down-regulated genes in BJAB-KSHV cells compared to BJAB cells. Asterisk (*) denotes FDR-p-value <0.05.

The top 10 up-regulated and top 10 down-regulated genes from the comparative group are provided (Fig 4D). Analysis of the RNA sequencing data for the differential gene expression in BJAB-KSHV cells compared to BJAB cells resulted in detection of 357 up-regulated transcripts (p≤0.001; FC ≤2 or ≥ 2). Similarly, 233 transcripts were observed down-regulated in BJAB-KSHV cells compared to BJAB cells (p≤0.001; FC ≤2 or ≥ 2). A volcano plot for the differentially expressed genes in BJAB-KSHV cells compared to BJAB cells is shown (Fig 5A). The top 10 up-regulated and top 10 down-regulated genes from the comparative group are provided (Fig 5B). To further corroborate the transcriptional profiles of CoCl_2_ induced hypoxia or the combinatorial effects of hypoxia and KSHV infection, a comparative analysis of RNA sequencing data between BJAB and BJAB-KSHV-CoCl_2_ was performed. The results revealed a more enhanced effect of CoCl_2_ on transcription of host genes. Compared to 2182 transcripts up-regulated in BJAB-CoCl_2_ cells, 2560 transcripts were up-regulated in BJAB-KSHV-CoCl_2_ cells (p≤0.001; FC ≤2 or ≥ 2). Similarly, an enhanced effect on down-regulation of transcripts was also observed in BJAB-KSHV-CoCl_2_ cells where a total of 2,143 transcripts were observed down-regulated (p≤0.001; FC ≤2 or ≥ 2), compared to only 1,882 genes in BJAB-CoCl_2_ cells. A volcano plot for the differential gene expression of BJAB vs. BJAB-KSHV-CoCl_2_ cells is shown in S3A Fig. The top 10 up-regulated and top 10 down-regulated genes from this comparative group is also provided in S3B Fig.

### Common targets are seen for KSHV infection and CoCl_2_-induced hypoxia with synergistic roles in transcriptional regulation of cellular genes

**Fig 6:**
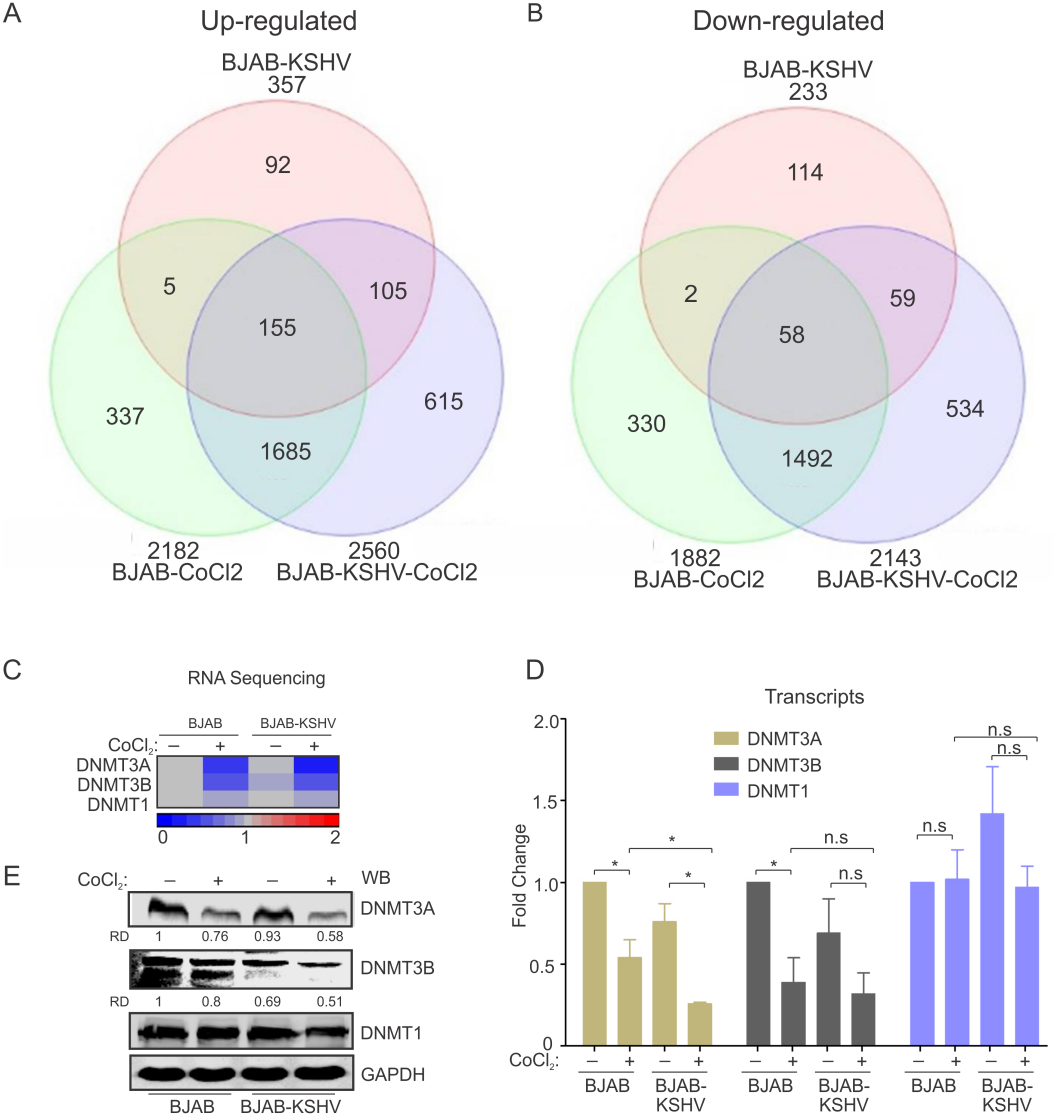
Common targets of hypoxia and KSHV infection. A list of genes from BJAB-KSHV, BJAB-CoCl_2_ and BJAB-KSHV-CoCl_2_ samples were generated with significant difference when compared to BJAB cells. The lists of gene names were added to the Partek software to identify common up-regulated or common down-regulated genes in these samples compared to BJAB cells. (A) Venn diagram showing common up-regulated factors targeted by hypoxia and KSHV. (B) Venn diagram showing common Down-regulated factors targeted by hypoxia and KSHV. (C) Intensity map for the fold change (RNA sequencing) of DNMT1, DNMT3A and DNMT3B. (D) Real time PCR validation of DNMTs expression. (E) Representative image of Western blot analysis for validation of DNMTs expression. Asterisk (*) indicates differences which are statistically significant (p≤0.05).

KSHV infection is known to stabilize HIF1α [16,20]. We wanted to determine which genes are common targets of KSHV infection, and CoCl_2_-induced hypoxia. We also analysed the data to identify synergistic activation or suppression activities linked to the combination of KSHV and hypoxia. A Venn diagram was prepared (using Partek software) for the differentially expressed genes (p ≤0.001; FC ≤2 or ≥ 2) in BJAB-CoCl_2_, BJAB-KSHV and BJAB-KSHV-CoCl_2_ cells compared to BJAB cells (Fig 6A). Among the 357 transcripts observed up-regulated in BJAB-KSHV cells and 2,182 transcripts up-regulated in BJAB-CoCl_2_ cells, 160 transcripts were common (Fig 6A). Similarly, among 233 down- regulated transcripts in BJAB-KSHV cell and 1,882 down-regulated transcripts in BJAB- CoCl_2_ cells, 60 transcripts were common (Fig 6A). Interestingly, 105 transcripts out of 357 up-regulated in BJAB-KSHV cells were specific for KSHV. These transcripts were also up-regulated in BJAB-KSHV-CoCl_2_ cells (Fig 6A). Among the 233 down-regulated genes in BJAB-KSHV cells, 59 were observed to be specific for KSHV. These genes were also down-regulated in BJAB-KSHV-CoCl_2_ cells (Fig 6A). Intensity maps of common up-regulated and down-regulated genes are provided in S4 Fig. We wanted to know if transcription regulatory genes were common targets of CoCl_2_-induced hypoxia, and KSHV infection. Our analysis showed that the DNMTs, mainly DNMT3A and DNMT3B were two common targets for down-regulation induced by both hypoxia and KSHV infection. Real-time PCR analysis for these DNMTs followed by western blot analysis for protein levels showed similar results as observed from our RNA sequencing, although DNMT3A was clearly more dramatic in its suppression (Fig 6C, 6D and 6E).

### The KSHV-HIF1α axis modulates the metabolic pathway

**Fig 7:**
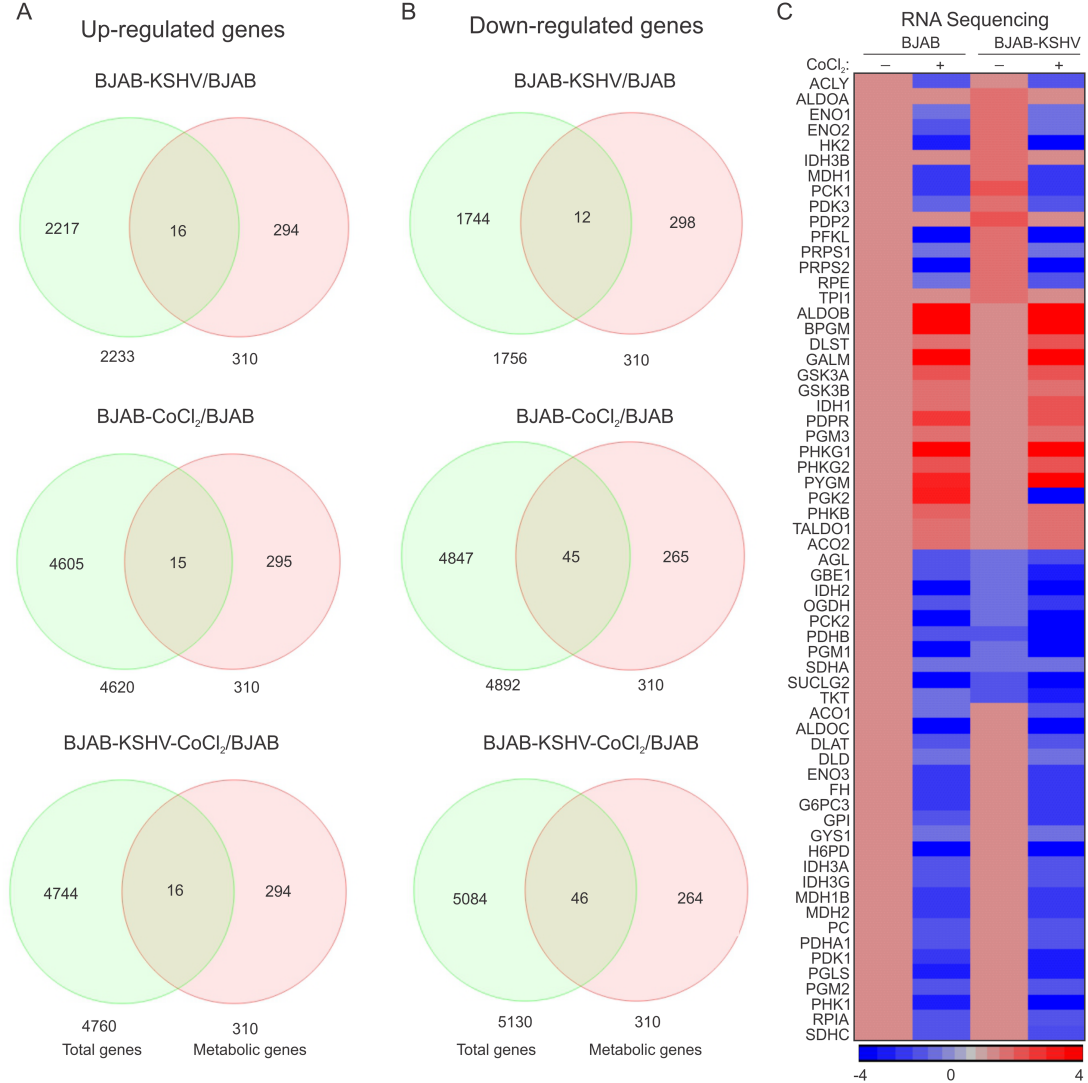
Differentially expressed metabolic genes in BJAB-KSHV and CoCl2 induced hypoxic BJAB/BJAB-KSHV cells compared to BJAB cells. (A) Venn diagram for up-regulated genes in BJAB-CoCl2/BJAB-KSHV/BJAB-KSHV-CoCl2 cells compared to BJAB cells. (B) Venn diagram for Down-regulated genes in BJAB-CoCl2/BJAB-KSHV/BJAB-KSHV-CoCl2 cells compared to BJAB cells. (C) Intensity map for the fold change of differentially expressed genes in BJAB-CoCl2/BJAB-KSHV/BJAB-KSHV-CoCl2 cells compared to BJAB cells. Non-significant fold change expression for genes in BJAB-CoCl_2_, BJAB-KSHV or BJAB-KSHV-CoCl_2_ cells compared to BJAB cells were considered 1 for generating intensity map.

**Fig 8:**
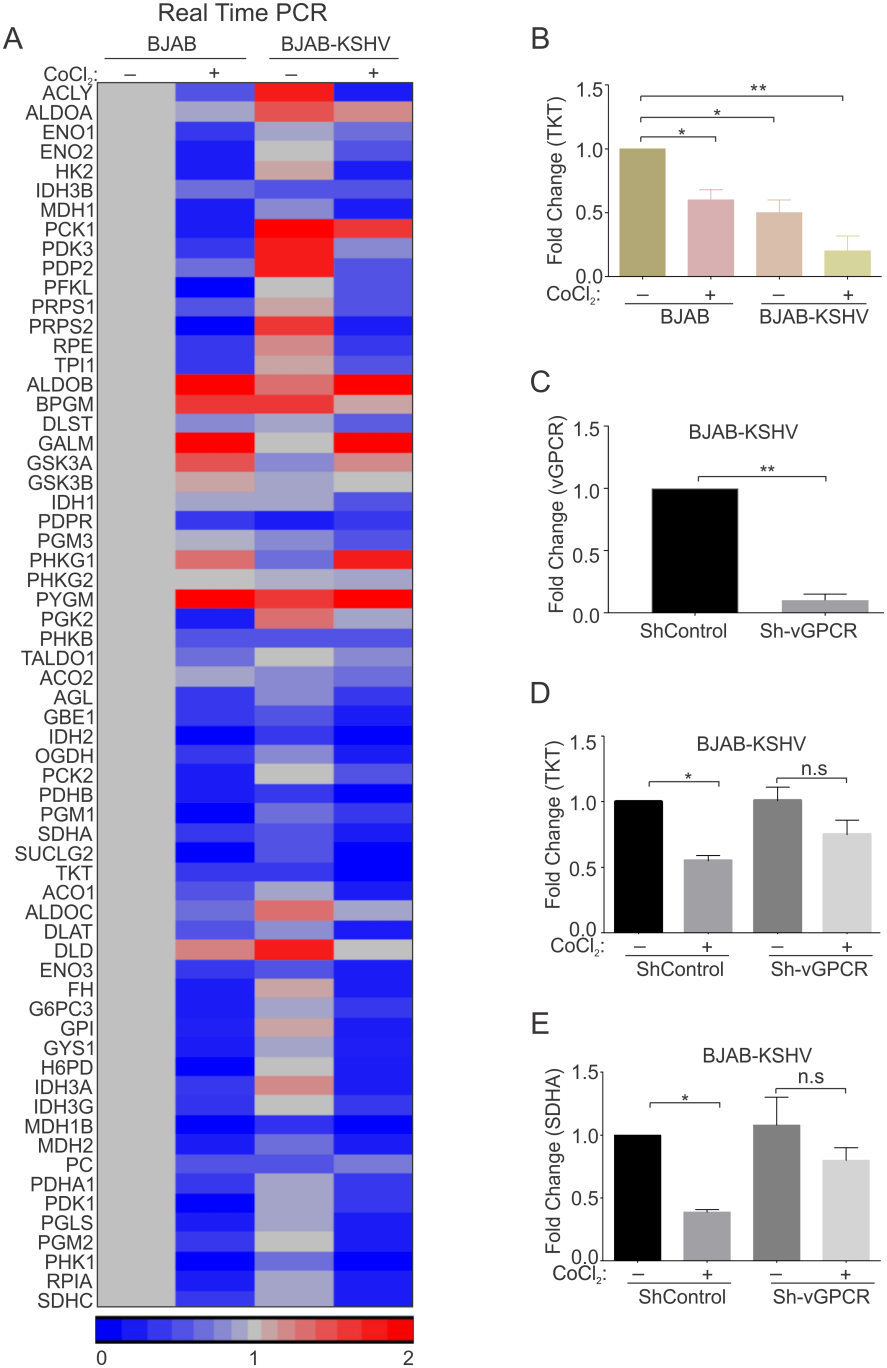
(A) Intensity map for the Real-time PCR fold change of differentially expressed genes in BJAB-CoCl2/BJAB-KSHV/BJAB-KSHV-CoCl2 cells compared to BJAB cells. A set of genes belonging to metabolic pathways were chosen to be validated by real time PCR. The difference in fold change of BJAB-CoCl2/BJAB-KSHV/BJAB-KSHV-CoCl2 cells when compared to BJAB cells were used to generate heat-intensity maps. The real-time PCR experiments were performed in duplicates, with an experimental repeat for each gene. (B) Real time PCR for TKT in BJAB and BJAB-KSHV cells grown in normoxia and CoCl_2_ induced hypoxia. (C) Real time PCR to determine expression of vGPCR in BJAB-KSHV cells infected with lentiviral based ShControl and Sh-vGPCR. (D) Real time PCR to determine expression of TKT in ShControl and Sh-vGPCR BJAB-KSHV cells grown under normoxic or CoCl_2_ induced hypoxic environment. (E) Real time PCR to determine expression of SDHA in ShControl and Sh-vGPCR BJAB-KSHV cells grown under normoxic or CoCl_2_ induced hypoxic environment. All real-time PCR assays were performed at least in triplicates, with experimental repeat for each gene. Asterisk (*) indicates differences which are statistically significant (* p≤0.05, **≤0.01).

310 genes involved in glucose, fatty acids and amino acids metabolism were identified by reviewing genes involved in these processes (S6 Table). This list was used to identify differentially expressed genes in each group when compared to BJAB cells. To optimize the number of genes differentially expressed in BJAB-KSHV, BJAB–CoCl_2_ or BJAB-KSHV-CoCl_2_, the analysis stringency was maintained to allow for statistical significance (p<0.05). A Venn diagram was created for the common genes from the 310 metabolic regulated genes which were differentially expressed (up-regulated or down-regulated) in each group above. Compared to BJAB cells, a total of 16 metabolic genes were up-regulated in BJAB-KSHV cells (Fig 7A). These up-regulated genes predominantly belonged to either glycolysis or the pentose phosphate pathway (ALDOA, ENO1, ENO2, HK2, PDK3, PDP2, PFKL and PGK1, PRPS1, PRPS2 and RPE), which supports a direct role for KSHV-infection in elevation of glycolysis. In addition, a subset of TCA cycle regulated genes ACLY, IDH3B, MDH1 and PCK1 were also up-regulated (Fig 7C). Interestingly, these genes were exclusively restricted to the KSHV-positive background, and were not up-regulated in cells grown under CoCl_2_-induced hypoxic condition (Fig 7A & 7C). Interestingly, in cells with elevated HIF1α due to CoCl_2_ treatment, activation of a different subset of metabolic genes from the glycolysis and TCA cycle pathways (15 genes for BJAB-CoCl_2_ and 16 genes for BJAB-KSHV-CoCl_2_) were identified. The up-regulated genes due to CoCl_2_ treatment were ALDOA, ALDOB, BPGM, PDPR and PGM3 (glycolysis) and DLST and IDH1 (TCA cycle) (Fig 7A & 7C). In addition, a set of glycogen synthesis genes (GSK3A, GSK3B, PHKG1, PHKG2 and PYGM) were also observed to be induced in CoCl_2_ treated cells (Fig 7A & 7C). Interestingly, HIF1α stabilization due to CoCl_2_ treatment appeared to be dominant over KSHV infection. The expressions of these up-regulated genes were similar in both BJAB and BJAB-KSHV cells treated with CoCl_2_ (Fig 7A & 7C). A second set of evidence showing glycolytic up-regulation by KSHV infection, or CoCl_2_-induced HIF1α was visible from the set of down-regulated genes in the TCA cycle (IDH2, PDHB, SDHA and SUCLG2), and glycogen metabolism (AGL, GB1, PCK2 and PGM1) (Fig 7B). These genes were down-regulated in both KSHV alone, and CoCl_2_ alone, in addition to their combination. CoCl_2_ treatment in fact showed an additional set of genes which were down-regulated compared to BJAB or BJAB-KSHV cells (Fig 7B & 7C). The differentially expressed genes observed by RNA sequencing were further validated by real time PCR (Fig 8A), using gene specific primers (S5 Table). Among the metabolic genes, Transketolase (TKT) and Succinate dehydrogenase subunit A (SDHA) were down-regulated by either KSHV-infection or CoCl_2_ treatment. Interestingly, the expression of both TKT and SDHA were suppressed by KSHV infection and HIF1α stabilization (Fig 8A and 8B). As vGPCR over-expression was associated with global transcriptional regulation and generation of reactive oxygen species (ROS) [27], we hypothesized that expression of these genes can be a consequence of induced vGPCR in the hypoxic environment. To confirm the role of vGPCR in the down-regulation of TKT and SDHA, vGPCR knock down BJAB-KSHV cells were generated by lentivirus based transduction (Fig 8C). Real-time expression of TKT and SDHA was analyzed in Sh-vGPCR BJAB-KSHV cells grown under normoxia or CoCl_2_ induced hypoxic conditions. The results showed clear involvement of vGPCR in regulating expression of these genes. ShControl cells showed a significant down-regulation of both TKT and SDHA expression in the hypoxic environment, however, Sh-vGPCR cells did not show any significant down-regulation (Fig 8D & 8E). Importantly, we did not observe any strong up-regulation of TKT and SDHA in hypoxia. However, in Sh-vGPCR cells their expression was definitely increased compared to wild type KSHV indicating a role in transcription regulation under hypoxic conditions.

### The vGPCR null KSHV is incompetent for metabolic changes in hypoxic environment

**Fig 9:**
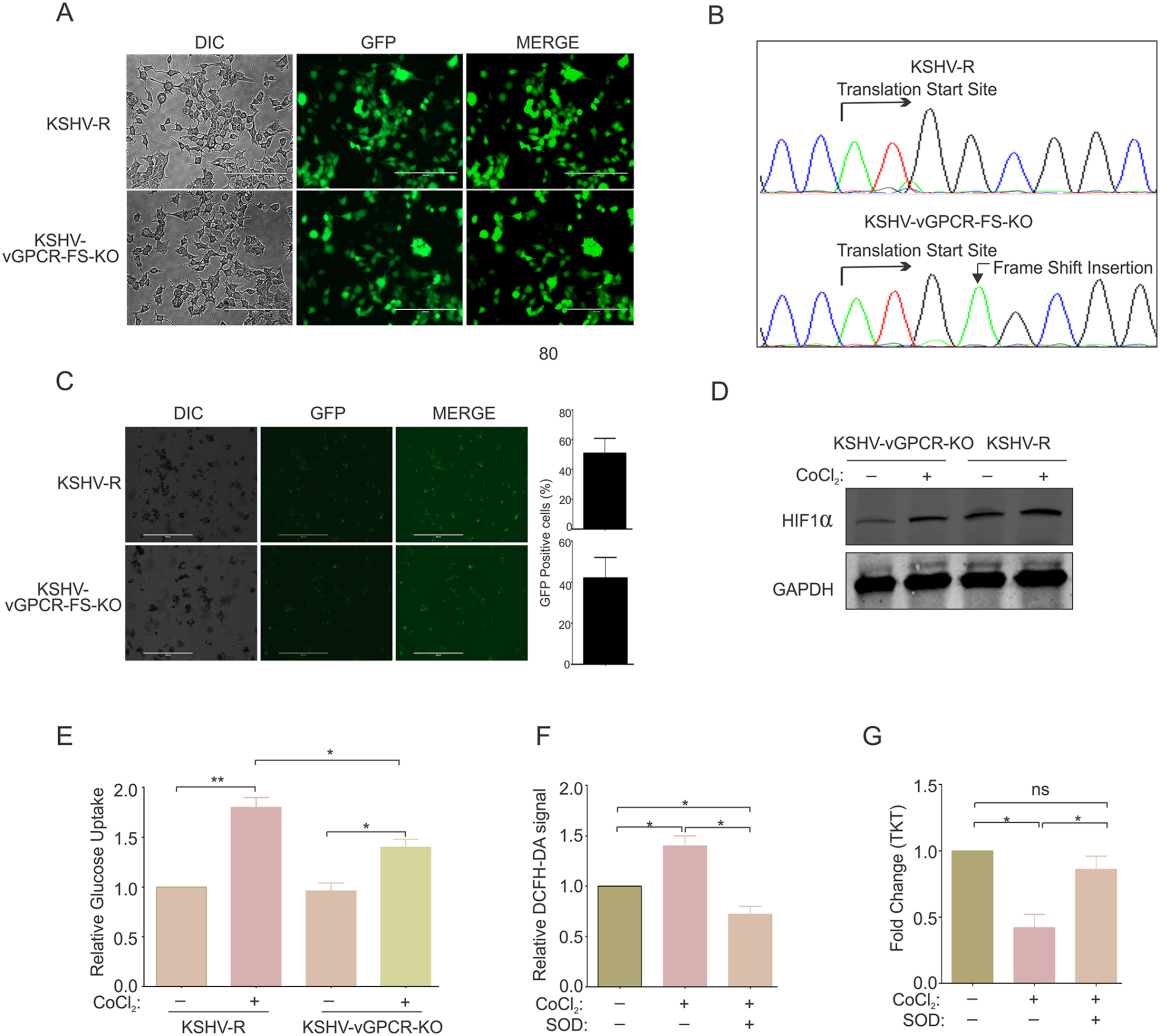
KSHV vGPCR knock out virus showed a loss in metabolic changes due to hypoxia induction compared to the wild type revertant. (A) Generation of HEK293T-BAC36-KSHV-vGPCR-frame shift knockout and recovered cells. BAC36-KSHV-vGPCR-frame shift knockout or reverted bacmids were electroporated in HEK293T cells and cells were selected with hygromycin (200μg/ml). (B) Confirmation of vGPCR-frame shift knockout. Genomic DNA from HEK293T-BAC36-KSHV-vGPCR-frame shift knockout or reverted cells were isolated followed by amplification of KSHV genomic region encompassing the vGPCR ORF. Arrow on the electropherogram shows translation start site and frame shift insertion. (C) Infection of PBMCs with vGPCR frame shift knock out and wild type revertant virion. The infection was monitored by GFP signals. Bar diagram represents percent infected cells empirically calculated by counts of GFP signals. (D) Confirmation of induction of hypoxia in CoCl_2_ treated PBMCs infected with vGPCR frame shift knock out and wild type revertant KSHV virion. KSHV infected cells were treated with CoCl2 for 48 hours followed by protein isolation and western blot against HIF1α. (E) Relative glucose uptake by PBMCs infected with wild type (revertant) or vGPCR frame shift knockout KSHV and grown under CoCl_2_ induced hypoxia. (F) Estimation of reactive oxygen species in PBMCs infected with KSHV with or without Superoxide dismutase treatment. (G) Real time PCR for expression of TKT in KSHV infected PBMCs with or without treatment with ROS scavenger SOD. Asterisk (*) indicates differences which are statistically significant (* p≤0.05, **≤0.01).

To further corroborate a role for KSHV-encoded vGPCR in metabolic changes observed in the hypoxic environment, we investigated whether vGPCR knockout cells showed a reversal of the metabolic phenotype observed under hypoxia. The KSHV-bacmid clone containing vGPCR-Frame shift knock out mutant (KSHV-vGPCR-FS-KO) or vGPCR-Frame shift mutant reversed (KSHV-vGPCR-FS-R) KSHV [28] were transfected into HEK293T cells to generate stable lines. The transfected GFP positive cells were selected using hygromycin (Fig 9A). To confirm that the frame shift mutation and revertant was maintained, genomic DNA from stable cells were isolated and the KSHV region encompassing the insertion site, PCR amplified and sequenced. The electropherogram showing the sequencing results confirmed the frame shift insertion and reversion to wild type (Fig 9B). KSHV reactivation of the vGPCR-Frame shift mutant or revertant was induced by treatment with TPA and Butyric acid followed by standard virus purification. As expected, the vGPCR-Frame shift mutant showed a substantial decrease in lytic replication with significantly less yield in genome copies. To determine if vGPCR was a critical factor for the observed metabolic changes in hypoxia, PBMCs were infected with the vGPCR knockout and revertant KSHV virus and cells were subjected to hypoxic induction by treating with CoCl_2_. KSHV infection was monitored by GFP signal and the induction of hypoxia was confirmed by western blot to detect HIF1α (Fig 9D). The percentage of cells infected with KSHV was empirically calculated by counts of GFP signals in the cell population. The infection efficiency of PBMCs with KSHV was approximately 50%, although we observed slightly weaker GFP signal intensity from cells infected with the KSHV-vGPCR-FS-KO (Fig 9C). Cells were grown in normoxic, or CoCl_2_ induced hypoxia for 48 hours followed by media collection and measurement of glucose uptake. The results suggest a clear role for KSHV-encoded vGPCR in contributing to the metabolic changes. The PBMCs infected with the vGPCR kockout KSHV showed a significantly lower glucose uptake compared to the revertant (Wild type) KSHV infected cells (Fig 9E). As vGPCR has been shown to modulate transcriptional changes through the global signaling molecule i.e reactive oxygen species (ROS), we wanted to determine if vGPCR mediated ROS had any role in the transcriptional regulation of genes which were differentially expressed in our study. We first determined the levels of reactive oxygen species in PBMCs infected with revertant (wild type) KSHV treated with or without ROS scavenger, superoxide dismutase (SOD) by DCFH-DA staining (Fig 9E and 9F). As expected, the results showed a high level of reactive oxygen species in PBMCs infected by KSHV. The level was significantly lower in infected cells treated with SOD (Fig 9F). RNA was isolated from these cells and reversed transcribed. The expression of TKT was determined by real-time PCR of cDNA. The results showed a reversal in the expression pattern of TKT upon SOD treatment, suggesting a possible role of vGPCR mediated ROS in transcriptional regulation of cellular genes.

## Discussion

Similar to infection with most of oncogenic viruses, KSHV infection leads to stabilization of HIFs in host cells by either preventing its degradation or by up-regulating its expression at the transcription level [24,29–33]. The stabilized HIF1α alone or in conjugation with host and viral factors modulates several physiologic pathways supporting survival and growth of the infected cells [8,24]. Further, stabilization of hypoxia inducible factors due to viral infection only partially mimic the *in vitro* experimental methods of inducing hypoxia in cell culture by growing the cells either under low oxygen or chemical induction by Cobalt Chloride (CoCl_2_)/Deferoxamine mesylate (DFO) [34,35]. The stabilization of hypoxia inducible factor due to viral infection activated the HIF1α dependent pathways, whereas hypoxia due to low oxygen led to activation of several other energy associated pathways such as the AMPK dependent pathways [36–38].

Independent of the HIF1α stabilization mechanism, the interaction of stabilized hypoxia inducible factors with KSHV factors resulted in modulation of several pathways with impact on the host cell as well as the virus biology [16,18,39]. Hypoxia induces expression of the latency associated nuclear antigen (LANA), the key viral factor responsible for attachment of viral episomal DNA to the host chromosome [15,40]. On the other hand hypoxia is known to induce lytic replication of KSHV as well as enhancing the reactivation potential of known chemical inducers TPA and Butyric acid [16,18].

LANA can be described as a bonafide oncogenic protein with the ability to degrade cellular tumor suppressors as well as activate oncogenes [41–43]. Therefore, we would predict an enhanced tumorigenic state of KSHV infected cells in the hypoxic environment. However, KSHV-encoded reactivation and transcriptional activator (RTA) is also a downstream target of HIF1α and so allows for the possibility that KSHV-positive cells grown in a hypoxic environment will be more susceptible to lytic reactivation. Independent of cell destiny, both the latent or lytic pathways require enhanced metabolic activities for generating their required macromolecular components. However, the exact contributors to induction of the hypoxic phenotype to KSHV-positive cells have not been fully explored. What are the differences in utilization of physiological pathways in KSHV-negative and KSHV-positive cells grown in hypoxia has not been completely explored, particularly in B-cell lineages.

A huge challenge to investigating these questions is the limitation of available KSHV negative cell line controls. The comparative studies done previously for KSHV modulated pathways were performed by infecting cells of epithelial or endothelial origin while taking the parental cells as control[44]. However, for studies in B-cells, peripheral blood mononuclear cells were used [45]. Furthermore, the efficiency of KSHV infection remains a critical determinant in these studies [46].

In our current study, we used BJAB and BJAB-KSHV cells with the same genetic background [25], for comparative analysis of differential metabolic signatures and associated mechanisms due to the change in transcription profiles. Characterization of BJAB-KSHV cells showed that it can be used as a model B-cell line for our comparative analysis. We first investigated the metabolic behavior of BJAB and BJAB-KSHV cells grown in 1% oxygen or CoCl_2_-induced hypoxia. The results suggested an enhanced glucose dependency and a cancer cell metabolic phenotype of high lactate release in BJAB-KSHV cells growing in hypoxia due to both low oxygen, as well as CoCl_2_ treatment as compared to their counter KSHV negative BJAB cells. The observed changes were not exclusive to long-term infected cells. The initial infection of PBMCs with KSHV can also induce a similar pattern of changes when grown under hypoxic conditions. Interestingly, hypoxia induction due to low oxygen concentration induced a much higher glucose dependency and lactate release compared to CoCl_2_-induced hypoxia. Also, hypoxia due to low oxygen did not allow long-term survival of cells compared to hypoxia induced due to CoCl_2_. A higher rate of cell death after 48 hours was observed in culture. As hypoxia induction due to CoCl_2_ treatment shared more physiological relevance with stabilized HIF1α in KSHV positive cells at least in terms of cell survival, the RNA sequencing experiment performed on BJAB-KSHV cells growing in normoxia or CoCl_2_ induced hypoxia led to identification of novel HIF1α targets encoded by KSHV. One such target, vGPCR, is a constitutive homolog of cellular GPCR [24] and has been implicated in up-regulation of pathways common to most cancer cells including MAP-kinase, and the angiogenic pathways [24]. Although, vGPCR is considered a lytic gene for KSHV reactivation [47], its role in tumorigenesis is also reported due to its activation of on MEK/ERK [24,47]. In addition, vGPCR is also known to inhibit transcription of other KSHV-encoded lytic genes [48,49]. It can modulate global expression of host genes as its induced expression in cells is associated with global changes in signaling due to increased levels of ROS [27]. The slightly higher expression of KSHV-encoded ORF2 (homolog of cellular Dihydrofolate reductase) suggest a shift towards the anabolic pathway for nitrogenous base synthesis, which is a pre-requisite for cell transformation and viral reactivation. The induced expression of vGPCR and its correlation with enhanced glucose uptake, as well as its suppression in ShHIF1α cells provided hints towards its role in metabolic changes in KSHV infected cells in hypoxia. The results showing differential expression of KSHV-encoded transcripts also provided information about the half life and stability of these KSHV-encoded transcripts. For example, despite being transcribed from the same regulatory region, the transcript levels of LANA, vCyclin and vFLIP showed differential abundance, as well as a different pattern of expression under varying conditions. Though, it is now a well known fact that the transcript abundance not only depends on its transcriptional generation but also on the rate of its decay as a consequence of either its half life or regulation by non-coding RNAs, it would be interesting to explore the mechanism behind the differential abundance of vCyclin and vFLIP specifically under hypoxic conditions.

The analysis of global transcription changes in KSHV positive background and its similarity to those in KSHV negative cells grown in hypoxia suggested that HIF1α plays a role in KSHV infection in its associated pathology. Recently, a study designed to explore common transcriptional signatures for hypoxia and KSHV infection was performed using cells of different origins under low oxygen conditions. A convergence of targets due to KSHV infection and hypoxia was observed [50]. However, this study identified a limited number of genes differentially expressed under these conditions [50]. In combination with our data, we now provide a global picture of the role of hypoxia, KSHV infection, and their combinatorial effect on transcription of cellular genes regulated in the background of KSHV-infected cells. Further, it is possible that the role of HIF1α in B-cells, and other cell lineages including epithelial or endothelial cells may have differences in terms of transcripts or translated products. In B-cells, the oxygen partial pressure resembles that of blood supply. This is likely very different in solid tumors originated from epithelial or endothelial cells. In KSHV infected B-cells, induced HIF1α levels is mainly due to signaling modulated by KSHV-encoded antigens, whereas in KSHV-infected endothelial cells, the stabilization of HIF1α is likely due to the combined effects of signaling modulated by KSHV-encoded antigens in addition to low oxygen. In KSHV-infected endothelial cells, the hypoxic conditions may lead to activation of AMPK pathways due to low oxygen supply, which eventually affects ATP production through mitochondrial respiration and accumulation of AMP. The effect of induced HIF1α in B-cells may not activate the AMPK pathways. This study also provided information on the differences due to hypoxia induced due to low oxygen compared to CoCl_2_, especially in terms of cell survival and growth. An analysis of the effects on transcription of metabolic genes shows the up-regulation of the glycolytic pathway due to KSHV infection, and that induced hypoxia can affect the same pathways but though different targets. However, shut-down of specific cellular genes was similar in hypoxia and in KSHV infection.

While investigating possible cellular factor(s) involved in transcriptional regulation, analysis of global modulators such as DNMTs showed a significant down-regulation in their expression. This was mainly for DNMT3A and 3B due to CoCl_2_ induced hypoxia (for both BJAB and BJAB-KSHV cells) as seen in our RNA sequencing data sets. The expression of both DNMT3A and 3B was low in KSHV infected cells, however, the differences between them were not significant. Validation of the RNA sequencing results of DNMTs expression by real-time PCR confirmed suppression by both hypoxia and KSHV infection on DNMT3A, and 3B expression. Further greater suppression of at least DNMT3A was seen when hypoxia and KSHV infection were combined. The analysis of DNMTs expression is likely not the only explanation for the changes in expression of these large set of genes. However, it would be interesting to investigate the role of other global modulators including small non-coding RNAs on the expression of large set of genes in response to hypoxia or/and KSHV. These studies are ongoing with potential for elucidating additional mechanistic insights into the hypoxia-KSHV axis.

## Materials and methods

### Ethics statement

Peripheral blood mononuclear cells (PBMCs) from undefined and healthy donors were obtained from the Human Immunology Core (HIC), University of Pennsylvania. The Human Immunology Core maintains approved protocols of Institutional Review Board (IRB) in which a Declaration of Helsinki protocols were followed and each donor/patient gave written, informed consent.

### Cell culture, plasmid constructs, and generation of lentiviral particles

BJAB (KSHV-negative B-cell) cells [51] were obtained from Elliot Kieff (Harvard Medical School,Boston, MA) originally purchased from American Type Culture Collection (ATCC). KSHV-positive BJAB-KSHV cells [25] were obtained from Michael Lagunoff (University of Washington, Seattle, WA). The KSHV-positive lymphoma-derived BC3 cell line was obtained from the ATCC. BJAB, BJAB-KSHV and BC3 cells were grown at 37°C/5% CO_2_ in RPMI medium containing 7% bovine growth serum (BGS) and penicillin (100 units/ml)/ streptomycin (0.1mg/ml). BJAB-KSHV cells were maintained with additional selection using puromycin (2μg/ml). Human Embryonic Kidney cell line (HEK 293T) was obtained from Jon Aster (Brigham and Womens Hospital, Boston, MA) and grown in DMEM medium containing 5% BGS with antibiotics at the above concentration. Cobalt chloride stock (100mM) was prepared in water. Hypoxia was induced by adding CoCl_2_ at a final concentration of 100 μM or by growing the cells in a hypoxia chamber with 1% oxygen. ShControl and ShHIF1α lentiviruses were generated by transfection of HEK293T cells with transfer plasmids and third generation packaging and envelop plasmids as descried earlier [52]. In brief, HEK293T cells were grown in 100 mm cell culture dish at 40–60% confluency. 10 μg of transfer plasmids in combination with packaging and envelop plasmids were transfected by calcium phosphate method. After initial discard of culture medium containing transfection mix, the supernatants were collected between 24–96 hours at 12 hours intervals. The supernatants were filtered through a 0.45 μm syringe filter and lentiviruses were concentrated by ultracentrifugation at 23,500 rpm for 2 hours [45,52]. The pelleted lentiviruses were resuspended in 1ml of complete medium and frozen until used for transduction. Transduction was performed by mixing cells with resuspended lentiviral stock in the presence of 8μg/ml polybrene. 48 hours post transduction, cells were selected in the presence of 2μg/ml puromycin. The pCEFL-vGPCR [53] construct was a kind gift from Enrique A. Mesri (University of Miami Miller School of Medicine, Miami, FL). The vGPCR-Knock Out (vGPCR-KO, Frame shift Mutant), vGPCR-Knock Out Reversed Bacmid clones, Sh-vGPCR lentiviral and control plasmids [28] were provided by John Nicholas (John Hopkins Bloomberg School of Public Health, Baltimore, MD). Large scale maxiprep for vGPCR-Knock Out (Frame shift Mutant) and vGPCR-Knock Out Reversed Bacmids were prepared using Luria broath culture and Qiagen large construct kit (Qiagen Inc., Hilden, Germany). Electroporation of HEK-293T cells were done in 4mm cuvette on Biorad Gene Pulser Xcell electroporation system. The positive clones of HEK293T-BAC-KSHV-vGPCR-KO and HEK293T-BAC-KSHV-vGPCR-KO reversed were selected in 100 μg/ml hygromycin.

### KSHV virion preparation and infection of peripheral blood mononuclear cells

KSHV virion stocks were prepared from the KSHV positive BC3 cells as previously described [45]. In brief, KSHV reactivation was mediated by adding TPA (to a final concentration of 20ng/ml) and Butyric acid (final concentration 3mM) and the cells were placed in an incubator at 37°C/5% CO_2_ for 5 days. The cells and supernatant were pelleted down by centrifugation for 30 minutes at 3,000 rpm. The supernatant was filtered through a 0.45 micron syringe filter. The cell pellets were resuspended in 10 ml of 1X PBS followed by 3X freeze/thaw cycle. The lysed cells were again collected by centrifugation for 30 minutes at 3000 rpm and the supernatants were filtered through 0.45 micron syringe filter and pooled. The filtrates were subjected to ultracentrifugation at 23,500 rpm for 2 hours to collect the KSHV virions. Infection of PBMCs was carried out in the presence of 8 μg/ml polybrene as described earlier [45].

### DNA/RNA isolation, cDNA preparation, real time PCR, DNA sequencing and short tandem repeat (STR) profiling

RNA isolation was performed according to standard method of phenol chloroform extraction using TRizol reagent (Ambion, Grand Island, NY). 2μg of total RNA was used to synthesize cDNA by random priming method using Superscript cDNA synthesis kit (Applied Biosystems Inc., Foster City, CA). 1 μl of 10 times diluted cDNA was used for real-time PCR using Power SYBR green PCR reagent (Applied Biosystems Inc., Carlsbad, CA) using a Step One Plus or Quant Studio system (Applied Biosystems Inc., Carlsbad, CA). All real-time PCR assays were performed in duplicates, with one experimental repeats for each gene. Real-time PCR for a select set of genes were performed at least in triplicate. The differences in fold change were calculated by delta delta CT method using default parameter settings. DNA from cells were isolated using Blood & cell culture DNA isolation mini kit (Qiagen Inc., Hilden, Germany). Gel eluted PCR product was used for DNA sequencing (DNA sequencing facility, Department of Genetics, University of Pennsylvania) using both forward and reverse primers. Short tandem repeat (STR) profiling for BJAB and BJAB-KSHV cells was performed using GenePrint 10 kit (Promega Inc, Madison, WI) at the genomics core facility, Department of Genetics, University of Pennsylvania.

### Glucose uptake and lactate release estimation

Glucose concentration available in cell culture medium was estimated using the hexokinase measurement kit (Sigma Inc., St. Louis, MO). The amount of glucose uptake by cells was measured by subtracting the amount of available glucose from the total amount of glucose available in fresh medium. The value of glucose uptake was finally normalized per million of cells. The amount of lactate present in the medium was estimated using the lactate estimation kit (BioVision Inc., Milpitas, CA). In brief, a standard curve for the known amount of lactate was created. A pilot experiment was performed using 1 μl and 10 μl of 1:10 times diluted cell culture medium from control or treated cells to estimate the range of lactate released.

### Reactive oxygen species determination

Intracellular reactive oxygen species was determined by fluorescence of the cell permeable dye DCFH-DA. DCFH-DA stock solution at a concentration of 5 mM was prepared in DMSO. In brief, cells were stained with 5μM DCFH-DA for 30 minutes in complete media at 37°C in cell culture incubator followed by collection of cells at 1500 rpm for 5 minutes. The cells were counted and equal numbers were used to determine fluorescence on a micro plate reader (Molecular Devices, Sunnyvale, CA).

### Western blot and confocal microscopy

Cell lysates were separated on SDS-polyacrylamide gels followed by wet transfer to nitrocellulose membrane. 5% skimmed milk was used for blocking at room temperature for 1 hour with gentle shaking. Primary antibody against HIF1α (Santa Cruz Biotechnology, Dallas, TX and Novus Biosciences, Littleton, CO), GAPDH (Novus Biosciences, Littleton, CO), DNMT3A and DNMT3B (Abcam, Cambridge, MA) were incubated overnight at 4°C with gentle shaking followed by washing with TBST 3-times (5 minutes intervals). Probing with IR conjugated secondary antibodies was performed at room temperature for 1 hour followed by washing with TBST. Membranes were scanned using an Odyssey scanner (LI-COR Inc., Lincoln, NE) for detection of bands. For confocal microscopy, 25,000 cells were semi-dried on 8-well glass slides followed by fixation in 4% paraformaldehyde. Combined permealization and blocking was performed in 1XPBS containing 0.3% Triton X-100 and 5% goat serum followed by washing with 1X PBS. Anti-LANA antibody (purified ascites) was diluted in PBS containing 1% BSA and 0.3% triton X-100 and was incubated overnight at 4°C. Slides were washed with PBS followed by incubation with Alexa448 conjugated anti-mouse secondary antibody. DAPI staining was performed for 15 minutes at room temperature followed by washing and mounting. Images were captured by confocal microscope (Olympus, Lambertville, NJ).

### Sample preparation, RNA sequencing and data analysis

Total RNA was isolated by standard phase extraction using TRizol reagent (Ambion Inc., Grand Island, NY). Isolated RNA was analyzed for quantity and quality using a Bio-photometer (Eppendorf Inc., Hamburg, Germany). Samples for RNA sequencing were prepared using Illumina RNA sequencing sample prep kit. The indexed ready to run samples were run on Illumina platform at the University of Washington (Core services). The reads for sequencing data were aligned with KSHV and the human genome. All the RNA sequencing experiments were performed in duplicates. The fold change expression and statistical relevance of the differential gene expression was calculated by CLC bio software (Qiagen Inc., Hilden, Germany). The differential gene expression was represented by volcano plot using R-software. Intensity plots for the fold-change expression of a selected set of genes were created using Partek software (Partek Inc., St. Louis, MO).

## Supporting information

S1 FigCharacterization of BJAB-KSHV cells (A) GFP expression in BJAB-KSHV cells grown in 2μg/ml puromycin selection. (B) Amplification of 10 different regions from the genomic DNA of BJAB-KSHV cells. (C) Amplification of vCyclin from the cDNA template from BJAB-KSHV cells, NTC denotes no template control. (D) Estimation of relative glucose uptake in BJAB-KSHV cells grown in the presence or absence of puromycin (48 hours). (E) Real time PCR for the differential expression of HIF1α and VEGFA BJAB-KSHV cells grown in the presence or absence of puromycin (48 hours). (D) and (E) represent mean of three independent experiments. Asterisk (*) indicates differences which are statistically significant, * p≤0.05. (F) A pilot experiment to determine the available lactate in cell culture medium. Known concentration of purified lactate (0 nmol, 2 nmol, 4 nmol, 6 nmol, 8 nmol and 10 nmol) were used to generate standard curve by taking absorbance at 570 nm. 2 different volumes of fresh culture medium (1 μl and 10μl) and 1 μl of medium from grown cultures were used in the experiment to determine possible range of lactate in medium.(TIF)

S2 Fig Differential gene expression of KSHV-encoded genes in naturally infected KSHV positive BC3 cells grown under CoCl2 induced hypoxia: (A) Real-time expression of ORF6, ORF7, ORF8, ORF9, ORF10, ORF11, ORF18, ORF25 and ORF26. (B) Real-time expression of ORF27, ORF28, ORF30, ORF31, ORF32, ORF33, ORF34, ORF35 and ORF40. (C) Real-time expression of ORF44, ORF54, ORF56, ORF57, ORF63, ORF64, ORF69, ORFK8.1 and ORFK14. (D) Real time PCR for expression of vFLIP in ShCon and ShHif1α knockdown cells grown either in normoxia or CoCl_2_/1% O_2_ induced hypoxia. Lentivirus based transduction was used to generate ShControl and ShHIF1α knockdown cells in BC3. The stably infected cells were selected in puromycin for 3 weeks. The stably transduced BC3 ShControl and ShHIF1α knockdown cells (100% GFP positive cells) were used for RNA isolation and subsequent cDNA synthesis. Differential gene expression for vFLIP in ShCon and ShHIF1α knockdown cells grown either in normoxia or CoCl_2_/1% O_2_ induced hypoxia were determined by real time PCR using gene specific primers. Bar diagram represents mean of three independent experiments. Asterisk (*) indicates differences which are statistically significant, * p≤0.05.
(TIF)

S3 Fig Differential gene expression between BJAB-KSHV-CoCl_2_/BJAB cells. (A) Volcano plot for differential gene expression between BJAB-KSHV/BJAB cells. The differential gene expression between BJAB-CoCl2 and BJAB cells were calculated using CLC bio software and the volcano plot generated using R- software. (B) Top 10 up-regulated genes and top 10 down-regulated genes in BJAB-KSHV cells compared to BJAB cells. Asterisk (*) denotes statistical significance in term of FDR-p-value <0.05.
(TIF)

S4 Fig Intensity plot for the differential gene expression in BJAB-KSHV, BJAB-CoCl_2_, and BJAB-KSHV-CoCl_2_ cells as compared to BJAB cells. The differences in gene expression between BJAB-KSHV vs BJAB, BJAB-CoCl_2_ vs BJAB, and BJAB-KSHV-CoCl_2_ vs BJAB were calculated using CLC Bio software and the set of common genes between the three groups were dertermined using Partek software. (A) Intensity plot for up-regulated genes. (B) Intensity plot for down-regulated genes.
(TIF)

S1 Table List of primers used for the amplification of 10 different regions from the genomic DNA of BJAB-KSHV cells. 10 different sets of primers; set 1 (6–93; 88 bp), set 2 (15934–15119; 85 bp), set 3 (29599–29679; 80bp), set 4 (44659–44771; 111 bp), set 5 (59654–59771; 117 bp), set 6 (74785–74872; 87 bp), set 7 (89650–89732; 82 bp), set 8 (104644–104728; 84 bp), set 9 (119504–119598) and set 10 (126602–126697) were used to amplify KSHV genomic regions from BJAB-KSHV cells (Lower Panel). BJAB cells were also used as negative control (Upper Panel).
(DOCX)

S2 Table List and comparative analysis of short tandem repeat (STR) markers used to profile BJAB and BJAB-KSHV cells. (DOCX)

S3 Table List of primers used for validation of differentially expressed KSHV genes and DNMTs. (DOCX)

S4 Table List of primers used to amplify different HREs containing promoter regions. (DOCX)

S5 Table List of real time PCR primers used to validate RNA sequencing fold change results. (DOCX)

S6 Table List of genes used to screen the metabolic profiles from total gene pool. (DOCX)

## References

[1] Mesri EA, Cesarman E, Boshoff C (2010) Kaposi’s sarcoma and its associated herpesvirus. Nat Rev Cancer 10: 707–719. doi: 10.1038/nrc2888 20865011 10.1038/nrc2888 PMC4721662

[2] Ablashi DV, Chatlynne LG, Whitman JE Jr., Cesarman E (2002) Spectrum of Kaposi’s sarcoma-associated herpesvirus, or human herpesvirus 8, diseases. Clin Microbiol Rev 15: 439–464. doi: 10.1128/CMR.15.3.439-464.2002 12097251 10.1128/CMR.15.3.439-464.2002 PMC118087

[3] Dourmishev LA, Dourmishev AL, Palmeri D, Schwartz RA, Lukac DM (2003) Molecular genetics of Kaposi’s sarcoma-associated herpesvirus (human herpesvirus-8) epidemiology and pathogenesis. Microbiol Mol Biol Rev 67: 175–212, table of contents. doi: 10.1128/MMBR.67.2.175-212.2003 12794189 10.1128/MMBR.67.2.175-212.2003 PMC156467

[4] Delgado T, Carroll PA, Punjabi AS, Margineantu D, Hockenbery DM, et al (2010) Induction of the Warburg effect by Kaposi’s sarcoma herpesvirus is required for the maintenance of latently infected endothelial cells. Proc Natl Acad Sci U S A 107: 10696–10701. doi: 10.1073/pnas.1004882107 20498071 10.1073/pnas.1004882107 PMC2890792

[5] Delgado T, Sanchez EL, Camarda R, Lagunoff M (2012) Global metabolic profiling of infection by an oncogenic virus: KSHV induces and requires lipogenesis for survival of latent infection. PLoS Pathog 8: e1002866 doi: 10.1371/journal.ppat.1002866 22916018 10.1371/journal.ppat.1002866 PMC3420960

[6] Molloy S (2014) Viral infection: KSHV flicks the metabolic switch. Nat Rev Microbiol 12: 723 doi: 10.1038/nrmicro3377 25402354 10.1038/nrmicro3377

[7] Mushtaq M, Darekar S, Kashuba E (2016) DNA Tumor Viruses and Cell Metabolism. Oxid Med Cell Longev 2016: 6468342 doi: 10.1155/2016/6468342 27034740 10.1155/2016/6468342 PMC4789518

[8] Ma T, Patel H, Babapoor-Farrokhran S, Franklin R, Semenza GL, et al (2015) KSHV induces aerobic glycolysis and angiogenesis through HIF-1-dependent upregulation of pyruvate kinase 2 in Kaposi’s sarcoma. Angiogenesis 18: 477–488. doi: 10.1007/s10456-015-9475-4 26092770 10.1007/s10456-015-9475-4 PMC4659376

[9] Liberti MV, Locasale JW (2016) The Warburg Effect: How Does it Benefit Cancer Cells? Trends Biochem Sci 41: 211–218. doi: 10.1016/j.tibs.2015.12.001 26778478 10.1016/j.tibs.2015.12.001 PMC4783224

[10] Vander Heiden MG, Cantley LC, Thompson CB (2009) Understanding the Warburg effect: the metabolic requirements of cell proliferation. Science 324: 1029–1033. doi: 10.1126/science.1160809 19460998 10.1126/science.1160809 PMC2849637

[11] Feng P, Park J, Lee BS, Lee SH, Bram RJ, et al (2002) Kaposi’s sarcoma-associated herpesvirus mitochondrial K7 protein targets a cellular calcium-modulating cyclophilin ligand to modulate intracellular calcium concentration and inhibit apoptosis. J Virol 76: 11491–11504. doi: 10.1128/JVI.76.22.11491-11504.2002 12388711 10.1128/JVI.76.22.11491-11504.2002 PMC136794

[12] Mesri EA, Feitelson MA, Munger K (2014) Human viral oncogenesis: a cancer hallmarks analysis. Cell Host Microbe 15: 266–282. doi: 10.1016/j.chom.2014.02.011 24629334 10.1016/j.chom.2014.02.011 PMC3992243

[13] Sanchez EL, Carroll PA, Thalhofer AB, Lagunoff M (2015) Latent KSHV Infected Endothelial Cells Are Glutamine Addicted and Require Glutaminolysis for Survival. PLoS Pathog 11: e1005052 doi: 10.1371/journal.ppat.1005052 26197457 10.1371/journal.ppat.1005052 PMC4510438

[14] Yogev O, Lagos D, Enver T, Boshoff C (2014) Kaposi’s sarcoma herpesvirus microRNAs induce metabolic transformation of infected cells. PLoS Pathog 10: e1004400 doi: 10.1371/journal.ppat.1004400 25255370 10.1371/journal.ppat.1004400 PMC4177984

[15] Veeranna RP, Haque M, Davis DA, Yang M, Yarchoan R (2012) Kaposi’s sarcoma-associated herpesvirus latency-associated nuclear antigen induction by hypoxia and hypoxia-inducible factors. J Virol 86: 1097–1108. doi: 10.1128/JVI.05167-11 22090111 10.1128/JVI.05167-11 PMC3255810

[16] Cai Q, Lan K, Verma SC, Si H, Lin D, et al (2006) Kaposi’s sarcoma-associated herpesvirus latent protein LANA interacts with HIF-1 alpha to upregulate RTA expression during hypoxia: Latency control under low oxygen conditions. J Virol 80: 7965–7975. doi: 10.1128/JVI.00689-06 16873253 10.1128/JVI.00689-06 PMC1563785

[17] Zhang L, Zhu C, Guo Y, Wei F, Lu J, et al (2014) Inhibition of KAP1 enhances hypoxia-induced Kaposi’s sarcoma-associated herpesvirus reactivation through RBP-Jkappa. J Virol 88: 6873–6884. doi: 10.1128/JVI.00283-14 24696491 10.1128/JVI.00283-14 PMC4054365

[18] Davis DA, Rinderknecht AS, Zoeteweij JP, Aoki Y, Read-Connole EL, et al (2001) Hypoxia induces lytic replication of Kaposi sarcoma-associated herpesvirus. Blood 97: 3244–3250. 11342455 10.1182/blood.v97.10.3244

[19] Recchia AG, De Francesco EM, Vivacqua A, Sisci D, Panno ML, et al (2011) The G protein-coupled receptor 30 is up-regulated by hypoxia-inducible factor-1alpha (HIF-1alpha) in breast cancer cells and cardiomyocytes. J Biol Chem 286: 10773–10782. doi: 10.1074/jbc.M110.172247 21266576 10.1074/jbc.M110.172247 PMC3060528

[20] Jham BC, Ma T, Hu J, Chaisuparat R, Friedman ER, et al (2011) Amplification of the angiogenic signal through the activation of the TSC/mTOR/HIF axis by the KSHV vGPCR in Kaposi’s sarcoma. PLoS One 6: e19103 doi: 10.1371/journal.pone.0019103 21559457 10.1371/journal.pone.0019103 PMC3084756

[21] Cannon ML, Cesarman E (2004) The KSHV G protein-coupled receptor signals via multiple pathways to induce transcription factor activation in primary effusion lymphoma cells. Oncogene 23: 514–523. doi: 10.1038/sj.onc.1207021 14724579 10.1038/sj.onc.1207021

[22] Angelova M, Ferris M, Swan KF, McFerrin HE, Pridjian G, et al (2014) Kaposi’s sarcoma-associated herpesvirus G-protein coupled receptor activates the canonical Wnt/beta-catenin signaling pathway. Virol J 11: 218 doi: 10.1186/s12985-014-0218-8 25514828 10.1186/s12985-014-0218-8 PMC4304609

[23] Martin D, Galisteo R, Ji Y, Montaner S, Gutkind JS (2008) An NF-kappaB gene expression signature contributes to Kaposi’s sarcoma virus vGPCR-induced direct and paracrine neoplasia. Oncogene 27: 1844–1852. doi: 10.1038/sj.onc.1210817 17934524 10.1038/sj.onc.1210817

[24] Sodhi A, Montaner S, Patel V, Zohar M, Bais C, et al (2000) The Kaposi’s sarcoma-associated herpes virus G protein-coupled receptor up-regulates vascular endothelial growth factor expression and secretion through mitogen-activated protein kinase and p38 pathways acting on hypoxia-inducible factor 1alpha. Cancer Res 60: 4873–4880. 10987301

[25] Chen L, Lagunoff M (2005) Establishment and maintenance of Kaposi’s sarcoma-associated herpesvirus latency in B cells. J Virol 79: 14383–14391. doi: 10.1128/JVI.79.22.14383-14391.2005 16254372 10.1128/JVI.79.22.14383-14391.2005 PMC1280215

[26] Dulak J, Loboda A, Jazwa A, Jozkowicz A (2007) Comment on "A novel role of hypoxia-inducible factor in cobalt chloride- and hypoxia-mediated expression of IL-8 chemokine in human endothelial cells". J Immunol 178: 4707; author reply 4707–4708. 17404246 10.4049/jimmunol.178.8.4707 PMC2096716

[27] Ma Q, Cavallin LE, Leung HJ, Chiozzini C, Goldschmidt-Clermont PJ, et al (2013) A role for virally induced reactive oxygen species in Kaposi’s sarcoma herpesvirus tumorigenesis. Antioxid Redox Signal 18: 80–90. doi: 10.1089/ars.2012.4584 22746102 10.1089/ars.2012.4584 PMC3503473

[28] Sandford G, Choi YB, Nicholas J (2009) Role of ORF74-encoded viral G protein-coupled receptor in human herpesvirus 8 lytic replication. J Virol 83: 13009–13014. doi: 10.1128/JVI.01399-09 19793819 10.1128/JVI.01399-09 PMC2786835

[29] Cuninghame S, Jackson R, Zehbe I (2014) Hypoxia-inducible factor 1 and its role in viral carcinogenesis. Virology 456–457: 370–383. doi: 10.1016/j.virol.2014.02.027 24698149 10.1016/j.virol.2014.02.027

[30] Nakamura M, Bodily JM, Beglin M, Kyo S, Inoue M, et al (2009) Hypoxia-specific stabilization of HIF-1alpha by human papillomaviruses. Virology 387: 442–448. doi: 10.1016/j.virol.2009.02.036 19321184 10.1016/j.virol.2009.02.036 PMC2674135

[31] Morinet F, Parent M, Bergeron C, Pillet S, Capron C (2015) Oxygen and viruses: a breathing story. J Gen Virol 96: 1979–1982. doi: 10.1099/vir.0.000172 25934794 10.1099/vir.0.000172

[32] Mazzon M, Peters NE, Loenarz C, Krysztofinska EM, Ember SW, et al (2013) A mechanism for induction of a hypoxic response by vaccinia virus. Proc Natl Acad Sci U S A 110: 12444–12449. doi: 10.1073/pnas.1302140110 23836663 10.1073/pnas.1302140110 PMC3725076

[33] Wakisaka N, Kondo S, Yoshizaki T, Murono S, Furukawa M, et al (2004) Epstein-Barr virus latent membrane protein 1 induces synthesis of hypoxia-inducible factor 1 alpha. Mol Cell Biol 24: 5223–5234. doi: 10.1128/MCB.24.12.5223-5234.2004 15169887 10.1128/MCB.24.12.5223-5234.2004 PMC419879

[34] Wu D, Yotnda P (2011) Induction and testing of hypoxia in cell culture. J Vis Exp. 10.3791/2899 PMC3217626 21860378

[35] Kim KS, Rajagopal V, Gonsalves C, Johnson C, Kalra VK (2006) A novel role of hypoxia-inducible factor in cobalt chloride- and hypoxia-mediated expression of IL-8 chemokine in human endothelial cells. J Immunol 177: 7211–7224. 17082639 10.4049/jimmunol.177.10.7211

[36] Mungai PT, Waypa GB, Jairaman A, Prakriya M, Dokic D, et al (2011) Hypoxia triggers AMPK activation through reactive oxygen species-mediated activation of calcium release-activated calcium channels. Mol Cell Biol 31: 3531–3545. doi: 10.1128/MCB.05124-11 21670147 10.1128/MCB.05124-11 PMC3165558

[37] Papandreou I, Lim AL, Laderoute K, Denko NC (2008) Hypoxia signals autophagy in tumor cells via AMPK activity, independent of HIF-1, BNIP3, and BNIP3L. Cell Death Differ 15: 1572–1581. doi: 10.1038/cdd.2008.84 18551130 10.1038/cdd.2008.84

[38] Ziello JE, Jovin IS, Huang Y (2007) Hypoxia-Inducible Factor (HIF)-1 regulatory pathway and its potential for therapeutic intervention in malignancy and ischemia. Yale J Biol Med 80: 51–60. 18160990 PMC2140184

[39] Wei F, Gan J, Wang C, Zhu C, Cai Q (2016) Cell Cycle Regulatory Functions of the KSHV Oncoprotein LANA. Front Microbiol 7: 334 doi: 10.3389/fmicb.2016.00334 27065950 10.3389/fmicb.2016.00334 PMC4811921

[40] Juillard F, Tan M, Li S, Kaye KM (2016) Kaposi’s Sarcoma Herpesvirus Genome Persistence. Front Microbiol 7: 1149 doi: 10.3389/fmicb.2016.01149 27570517 10.3389/fmicb.2016.01149 PMC4982378

[41] Cai QL, Knight JS, Verma SC, Zald P, Robertson ES (2006) EC5S ubiquitin complex is recruited by KSHV latent antigen LANA for degradation of the VHL and p53 tumor suppressors. PLoS Pathog 2: e116 doi: 10.1371/journal.ppat.0020116 17069461 10.1371/journal.ppat.0020116 PMC1626105

[42] Friborg J Jr., Kong W, Hottiger MO, Nabel GJ (1999) p53 inhibition by the LANA protein of KSHV protects against cell death. Nature 402: 889–894. doi: 10.1038/47266 10622254 10.1038/47266

[43] Akram N, Imran M, Noreen M, Ahmed F, Atif M, et al (2017) Oncogenic Role of Tumor Viruses in Humans. Viral Immunol 30: 20–27. doi: 10.1089/vim.2016.0109 27830995 10.1089/vim.2016.0109

[44] Sychev ZE, Hu A, DiMaio TA, Gitter A, Camp ND, et al (2017) Integrated systems biology analysis of KSHV latent infection reveals viral induction and reliance on peroxisome mediated lipid metabolism. PLoS Pathog 13: e1006256 doi: 10.1371/journal.ppat.1006256 28257516 10.1371/journal.ppat.1006256 PMC5352148

[45] Jha HC, Lu J, Verma SC, Banerjee S, Mehta D, et al (2014) Kaposi’s sarcoma-associated herpesvirus genome programming during the early stages of primary infection of peripheral blood mononuclear cells. MBio 5. 10.1128/mBio.02261-14 PMC4271552 25516617

[46] Dollery SJ, Santiago-Crespo RJ, Kardava L, Moir S, Berger EA (2014) Efficient infection of a human B cell line with cell-free Kaposi’s sarcoma-associated herpesvirus. J Virol 88: 1748–1757. doi: 10.1128/JVI.03063-13 24257608 10.1128/JVI.03063-13 PMC3911588

[47] Xie J, Ajibade AO, Ye F, Kuhne K, Gao SJ (2008) Reactivation of Kaposi’s sarcoma-associated herpesvirus from latency requires MEK/ERK, JNK and p38 multiple mitogen-activated protein kinase pathways. Virology 371: 139–154. doi: 10.1016/j.virol.2007.09.040 17964626 10.1016/j.virol.2007.09.040 PMC2239004

[48] Cannon M, Cesarman E, Boshoff C (2006) KSHV G protein-coupled receptor inhibits lytic gene transcription in primary-effusion lymphoma cells via p21-mediated inhibition of Cdk2. Blood 107: 277–284. doi: 10.1182/blood-2005-06-2350 16150942 10.1182/blood-2005-06-2350 PMC1895347

[49] Bottero V, Sharma-Walia N, Kerur N, Paul AG, Sadagopan S, et al (2009) Kaposi sarcoma-associated herpes virus (KSHV) G protein-coupled receptor (vGPCR) activates the ORF50 lytic switch promoter: a potential positive feedback loop for sustained ORF50 gene expression. Virology 392: 34–51. doi: 10.1016/j.virol.2009.07.002 19640558 10.1016/j.virol.2009.07.002 PMC2747482

[50] Viollet C, Davis DA, Tekeste SS, Reczko M, Ziegelbauer JM, et al (2017) RNA Sequencing Reveals that Kaposi Sarcoma-Associated Herpesvirus Infection Mimics Hypoxia Gene Expression Signature. PLoS Pathog 13: e1006143 doi: 10.1371/journal.ppat.1006143 28046107 10.1371/journal.ppat.1006143 PMC5234848

[51] Menezes J, Leibold W, Klein G, Clements G (1975) Establishment and characterization of an Epstein-Barr virus (EBC)-negative lymphoblastoid B cell line (BJA-B) from an exceptional, EBV-genome-negative African Burkitt’s lymphoma. Biomedicine 22: 276–284. 179629

[52] Cai Q, Verma SC, Choi JY, Ma M, Robertson ES (2010) Kaposi’s sarcoma-associated herpesvirus inhibits interleukin-4-mediated STAT6 phosphorylation to regulate apoptosis and maintain latency. J Virol 84: 11134–11144. doi: 10.1128/JVI.01293-10 20719954 10.1128/JVI.01293-10 PMC2953196

[53] Bais C, Santomasso B, Coso O, Arvanitakis L, Raaka EG, et al (1998) G-protein-coupled receptor of Kaposi’s sarcoma-associated herpesvirus is a viral oncogene and angiogenesis activator. Nature 391: 86–89. doi: 10.1038/34193 9422510 10.1038/34193

