# Supplementary figures and images for: Metabolic reprogramming of Kaposi’s sarcoma associated herpes virus infected B-cells in hypoxia
Source: PLoS Pathog. 2018 May 10;14(5):e1007062. doi: 10.1371/journal.ppat.1007062 (PMC5963815; doi:10.1371/journal.ppat.1007062)

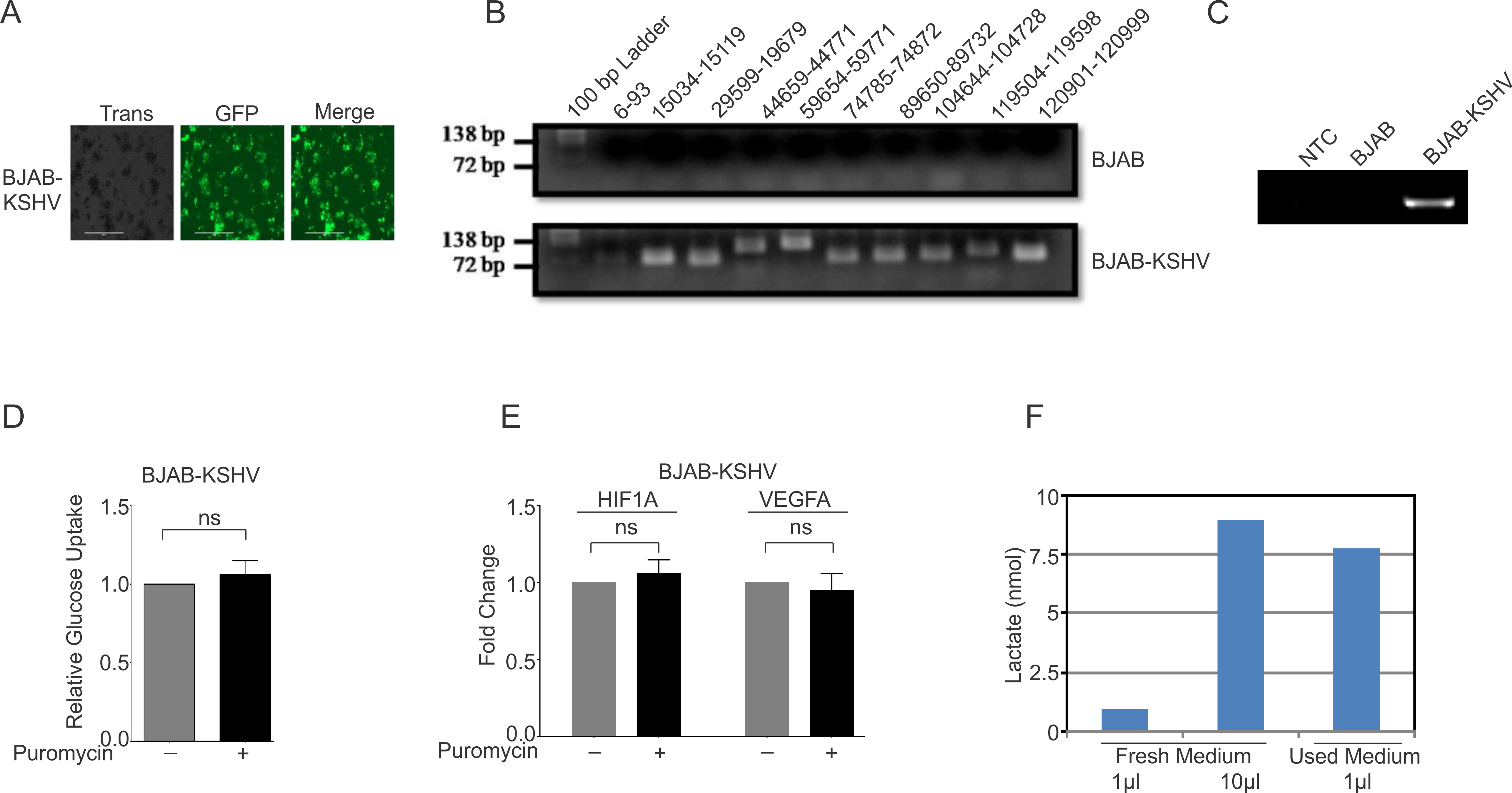

Supplement: S1 Fig — Characterization of BJAB-KSHV cells (A) GFP expression in BJAB-KSHV cells grown in 2μg/ml puromycin selection. (B) Amplification of 10 different regions from the genomic DNA of BJAB-KSHV cells. (C) Amplification of vCyclin from the cDNA template from BJAB-KSHV cells, NTC denotes no template control. (D) Estimation of relative glucose uptake in BJAB-KSHV cells grown in the presence or absence of puromycin (48 hours). (E) Real time PCR for the differential expression of HIF1α and VEGFA BJAB-KSHV cells grown in the presence or absence of puromycin (48 hours). (D) and (E) represent mean of three independent experiments. Asterisk (*) indicates differences which are statistically significant, * p≤0.05. (F) A pilot experiment to determine the available lactate in cell culture medium. Known concentration of purified lactate (0 nmol, 2 nmol, 4 nmol, 6 nmol, 8 nmol and 10 nmol) were used to generate standard curve by taking absorbance at 570 nm. 2 different volumes of fresh culture medium (1 μl and 10μl) and 1 μl of medium from grown cultures were used in the experiment to determine possible range of lactate in medium. (TIF) [file ppat.1007062.s001.tif]

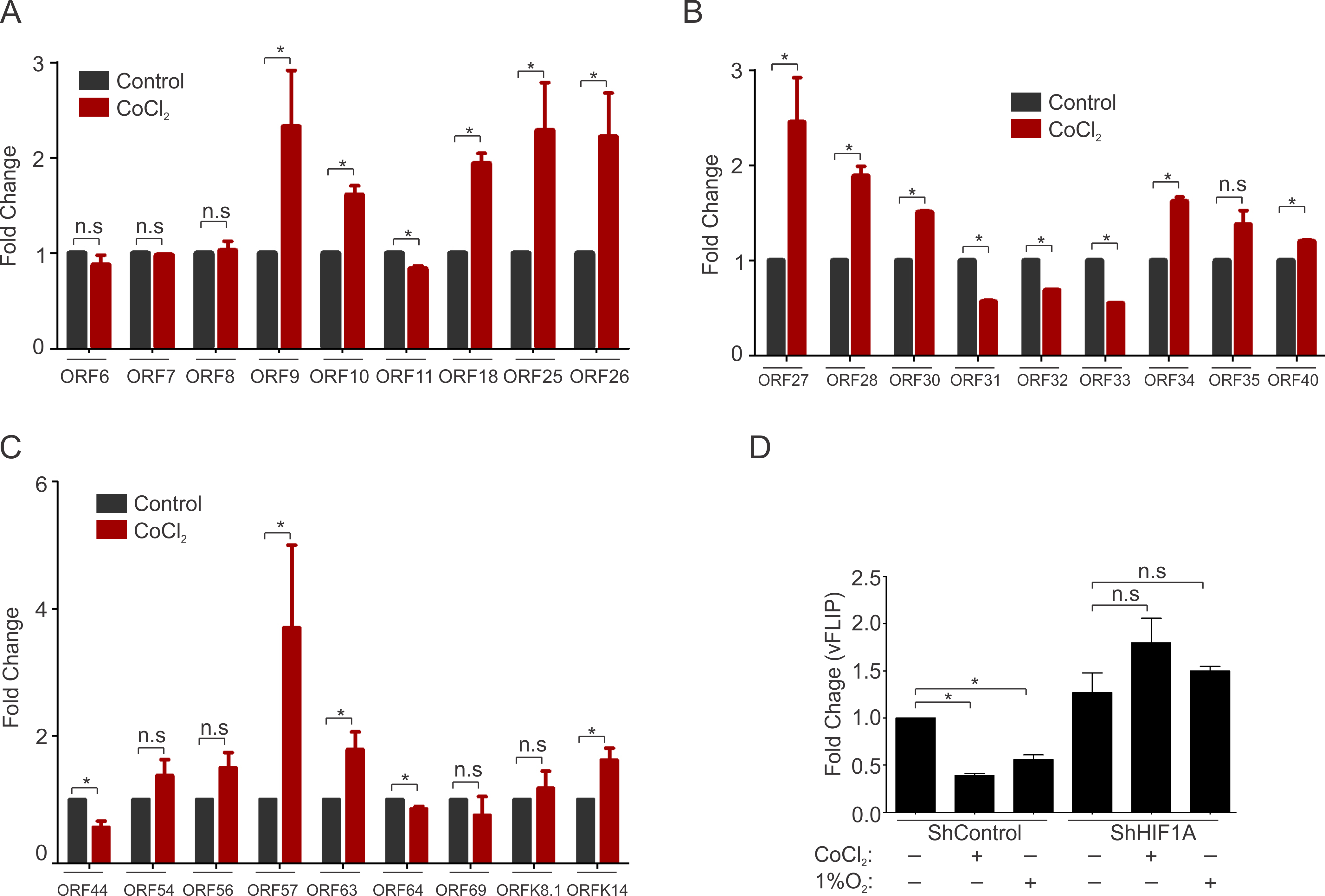

Supplement: S2 Fig — Differential gene expression of KSHV-encoded genes in naturally infected KSHV positive BC3 cells grown under CoCl2 induced hypoxia: (A) Real-time expression of ORF6, ORF7, ORF8, ORF9, ORF10, ORF11, ORF18, ORF25 and ORF26. (B) Real-time expression of ORF27, ORF28, ORF30, ORF31, ORF32, ORF33, ORF34, ORF35 and ORF40. (C) Real-time expression of ORF44, ORF54, ORF56, ORF57, ORF63, ORF64, ORF69, ORFK8.1 and ORFK14. (D) Real time PCR for expression of vFLIP in ShCon and ShHif1α knockdown cells grown either in normoxia or CoCl2/1% O2 induced hypoxia. Lentivirus based transduction was used to generate ShControl and ShHIF1α knockdown cells in BC3. The stably infected cells were selected in puromycin for 3 weeks. The stably transduced BC3 ShControl and ShHIF1α knockdown cells (100% GFP positive cells) were used for RNA isolation and subsequent cDNA synthesis. Differential gene expression for vFLIP in ShCon and ShHIF1α knockdown cells grown either in normoxia or CoCl2/1% O2 induced hypoxia were determined by real time PCR using gene specific primers. Bar diagram represents mean of three independent experiments. Asterisk (*) indicates differences which are statistically significant, * p≤0.05. (TIF) [file ppat.1007062.s002.tif]

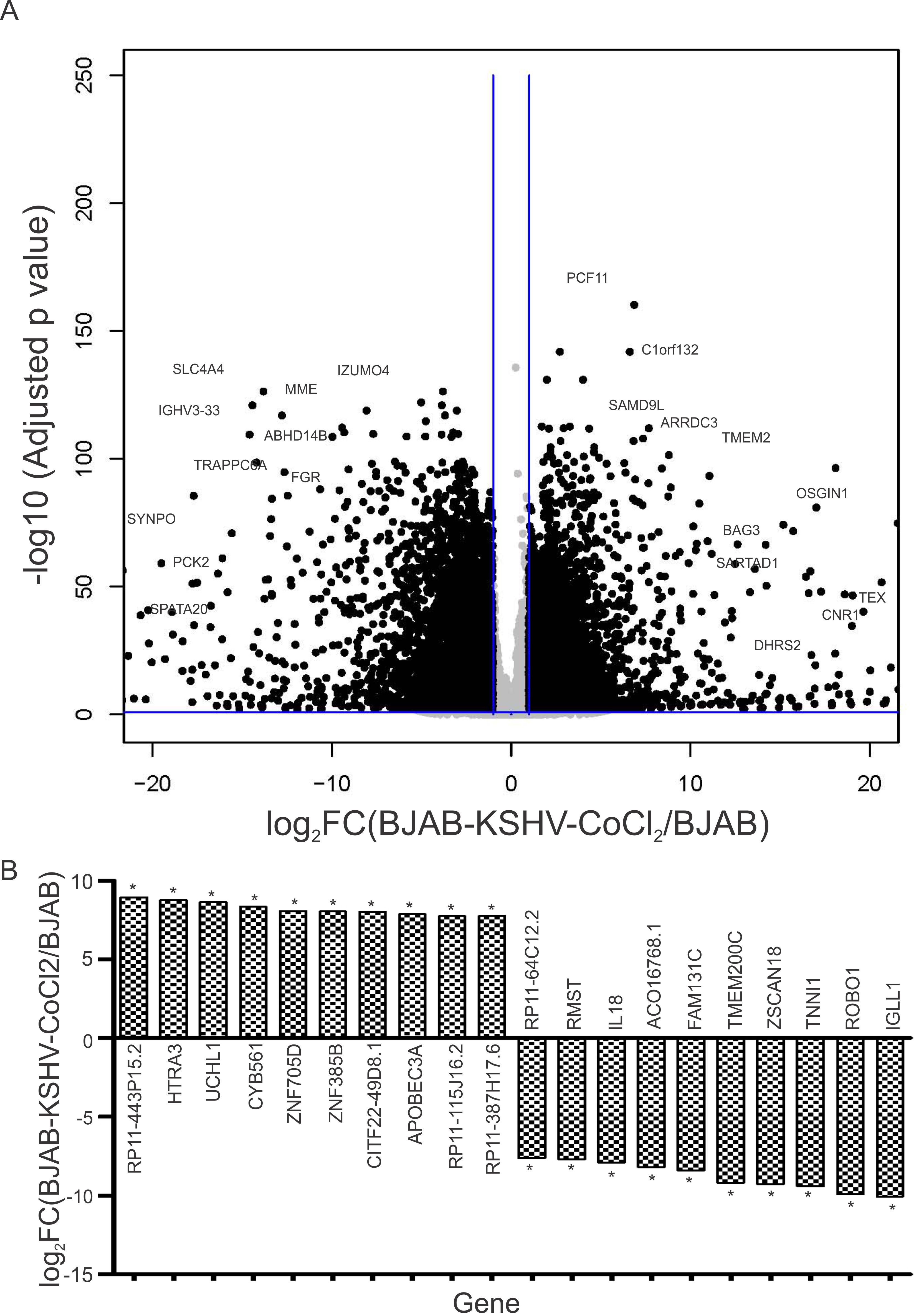

Supplement: S3 Fig — (A) Volcano plot for differential gene expression between BJAB-KSHV/BJAB cells. The differential gene expression between BJAB-CoCl2 and BJAB cells were calculated using CLC bio software and the volcano plot generated using R- software. (B) Top 10 up-regulated genes and top 10 down-regulated genes in BJAB-KSHV cells compared to BJAB cells. Asterisk (*) denotes statistical significance in term of FDR-p-value <0.05. (TIF) [file ppat.1007062.s003.tif]

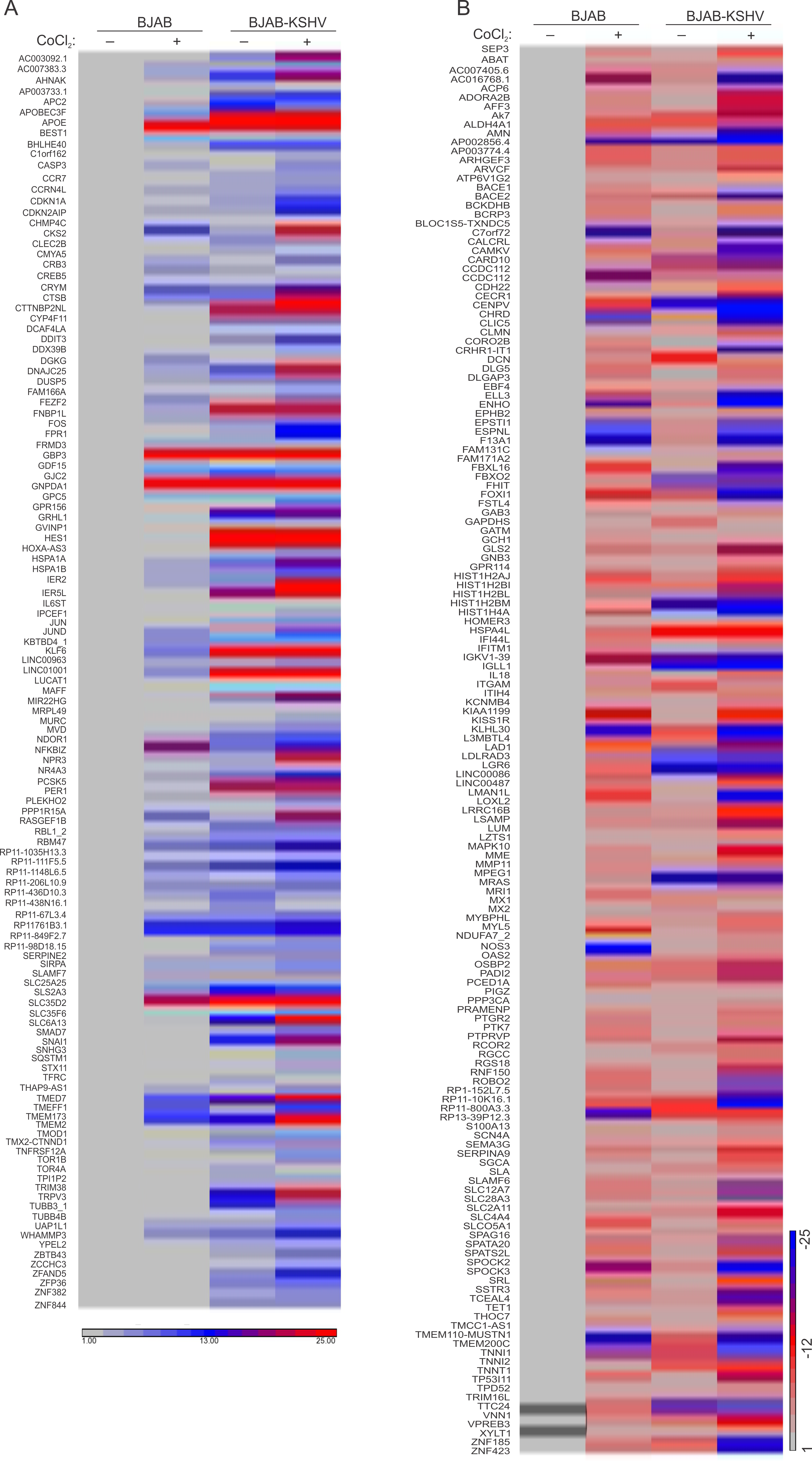

Supplement: S4 Fig — The differences in gene expression between BJAB-KSHV vs BJAB, BJAB-CoCl2 vs BJAB, and BJAB-KSHV-CoCl2 vs BJAB were calculated using CLC Bio software and the set of common genes between the three groups were dertermined using Partek software. (A) Intensity plot for up-regulated genes. (B) Intensity plot for down-regulated genes. (TIF) [file ppat.1007062.s004.tif]
