# Supplementary material for: Metabolic reprogramming of Kaposi’s sarcoma associated herpes virus infected B-cells in hypoxia
Source: PLoS Pathog. 2018 May 10;14(5):e1007062. doi: 10.1371/journal.ppat.1007062 (PMC5963815; doi:10.1371/journal.ppat.1007062)
Supplement: S1 Table — 10 different sets of primers; set 1 (6–93; 88 bp), set 2 (15934–15119; 85 bp), set 3 (29599–29679; 80bp), set 4 (44659–44771; 111 bp), set 5 (59654–59771; 117 bp), set 6 (74785–74872; 87 bp), set 7 (89650–89732; 82 bp), set 8 (104644–104728; 84 bp), set 9 (119504–119598) and set 10 (126602–126697) were used to amplify KSHV genomic regions from BJAB-KSHV cells (Lower Panel). BJAB cells were also used as negative control (Upper Panel). (DOCX) [file ppat.1007062.s005.docx]

**S1 Table:** List of primers used for the amplification of 10 different regions from the genomic DNA of BJAB-KSHV cells.

| Primer | KSHV Co-ordinates |  | Amplicon Size |  | Sequence (5’-3’) |  |
| --- | --- | --- | --- | --- | --- | --- |
| Set 1 | 6 – 93 |  | 88 bp |  | ATT TTGAAAGGCGGGGTTCTGC |  |
|  |  |  |  |  | GGGATGTCCAACAAGGTCTGAAG |  |
| Set 2 | 15934 – 15119 |  | 85 bp |  | TCCACCTCGCCACGAACGTATA |  |
|  |  |  |  |  | CCATGGACTCGAATGTCAGGAG |  |
| Set 3 | 29599 – 29679 |  | 80 bp |  | CCAGACGGCAAGGTTTTTATCC |  |
|  |  |  |  |  | TTGAGCTCTAGGCACGTTA |  |
| Set 4 | 44659 – 44771 |  | 111bp |  | GATGAACATGCCCCTCATTG |  |
|  |  |  |  |  | CCCCA ATGCGTACAGATGA |  |
| Set 5 | 59654 – 59771 |  | 117 bp |  | GTTATCAGTGACTGGATGTGGG |  |
|  |  |  |  |  | GATGTGGAGCATGCAACTGT |  |
| Set 6 | 74785 – 74872 |  | 87 bp |  | AAAGGGGGTGGTATTTCCTC |  |
|  |  |  |  |  | GGTATGTCCTTCATTCTGGGC |  |
| Set 7 | 89650 – 89732 |  | 82 bp |  | CATATCGAACTGTTCTGCCG |  |
|  |  |  |  |  | TCGCATACAGGGACATGAGC |  |
| Set 8 | 104644 - 104728 |  | 84 bp |  | TACCGCACCATTGTGTTCG |  |
|  |  |  |  |  | GATTTCGGTGATGGAGCATG |  |
| Set 9 | 119504 -119598 |  | 94 bp |  | AGTTCTAGGGATAGGGGCCA |  |
|  |  |  |  |  | AATCTCGTTCGAGCTAGGCG |  |
| Set 10 | 126602 - 126697 |  | 96bp |  | ACGGTTGGCGAAGTCACAT |  |
|  |  |  |  |  | AAAAGAAGGCTATCGTCCCC |  |
