# Supplementary material for: Metabolic reprogramming of Kaposi’s sarcoma associated herpes virus infected B-cells in hypoxia
Source: PLoS Pathog. 2018 May 10;14(5):e1007062. doi: 10.1371/journal.ppat.1007062 (PMC5963815; doi:10.1371/journal.ppat.1007062)
Supplement: S2 Table — (DOCX) [file ppat.1007062.s006.docx]

| STR Markers | BJAB | BJAB-KSHV | BJAB (ExPASy Bioinformatics Resource Portal Database) |
| --- | --- | --- | --- |
| D5S818 | 12,13 | 12,13 | 12,13 |
| D13S317 | 9,11 | 9,11 | 9,11 |
| D7S820 | 10,11 | 10,11 | 10,11 |
| D16S539 | 9,11 | 9,11 | 9,11 |
| vWA | 14,16 | 14,16 | 14,15(DSMZ)  14,16(Pubmed; 25877200) |
| TH01 | 7 | 7 | 7 |
| Amelogenein | X | X | X |
| TPOX | 6,9 | 6,9 | 6,9 |
| CSF1PO | 8,10 | 8,10 | 8,10 |

**S2 Table:** List and comparative analysis of Short Tandem Repeat (STR) markers used to profile BJAB and BJAB-KSHV cells.
