# Supplementary material for: Metabolic reprogramming of Kaposi’s sarcoma associated herpes virus infected B-cells in hypoxia
Source: PLoS Pathog. 2018 May 10;14(5):e1007062. doi: 10.1371/journal.ppat.1007062 (PMC5963815; doi:10.1371/journal.ppat.1007062)
Supplement: S3 Table — (DOCX) [file ppat.1007062.s007.docx]

**S3 Table:** Primers used for validation of differentially expressed KSHV genes and DNMTs

| Gene | Forward Primer (5'-3') | Reverse Primer (5'-3') |
| --- | --- | --- |
| vIRF1 | AGTGGGAAGACGAAGAGCGC | GCATGTTACCCCTTTCGCGG |
| vIRF2 | TAAGGACCGCCAATCGAGCC | CCTTCACATCCCTTGTATGGCC |
| vIRF3 | GGAATGCCTATCGAAGGGCC | ACCTGGCAAGCGTCTAAGGC |
| vIRF4 | GTTGCGTGGAACCAGGAGGG | TTTTAAACGGCGTTCGCACC |
| ORF2 | TTGCGGGGAGACATGAGGCG | CCGCCAATGGTCGACAGCCC |
| ORF4 | TCTCGTGACGGTGACATTGC | GGTTCAGACCACGTCCCACC |
| vGPCR | CGGGCAGGAGCGATAGATAT | GCCTGTGGAGATGATATTGGG |
| K1 | CTGGTATTGCAACGATACTCGGC | GCCCAGATTGTCCCACACAACTAA |
| K12 | ATGGATAGAGGCTTAACGGTGTTTG | TCAGTGCGCGCCCGTTGCAACTCG |
| vFLIP | TCCGATGGAGAGGCTAAGCG | GATGGTGGTTTGCCTGGTGC |
| ORF6 | ACAGTCTGGTGGCTGAACTTGG | CAAAGATGGGCCACGTGTCATA |
| ORF7 | TTGTATAGGATCGGGCAGTGCT | ACATATAGGTCCGGCCCAGGTA |
| ORF8 | GGTCCACGGACAAC TGTCTTA | CCATGCCCTGGAGAGTTCTTCT |
| ORF9 | TAAACCTGACCCACGTCCTCCA | TTTTTCTGACCGGCTCGGTGGA |
| ORF10 | TCCACCTCGCCACGAACGTATA | CCATGGACTCGAATGTCAGGAG |
| ORF11 | CACTGCATTCTACGCTTTTGGG | AATCAGCACGCTCGAGGAGCTT |
| ORF18 | GTGTGGAAGCTCGTGTACGA | AAGTGCATCCAGAGGCTGTC |
| ORF25 | CAGAGAAGGCAGTGTCCGTT | GGCGGTCTCCAGAAACTTAA |
| ORF26 | TACTCCAAAATATCGGCCGG | GCGCCCCATAAATGACACAT |
| ORF27 | GTTAACACGTCATGTGCAGGAG | GCAGATTTTCCCACTCAGTGTG |
| ORF28 | TATCAGGTCCTTCGCACCCT | TATTGTACGGTAGGGCTCCTGG |
| ORF30 | TTTCAGTCAACCCCTTAGGG | CAGCAGGCAGAGTCTTTCTGTT |
| ORF31 | GGGAAGACACTCACCGAATGTT | CACCGTAATCGTGCAGGAA |
| ORF32 | TGACGGGGTTGAAGCTCAGT | GTGCGTGGAGAAAGCCTAGA |
| ORF33 | CGAGCCAAACAAAAGGGTTC | TTAGTTCATACCTCCCAGGTGG |
| ORF34 | GTTCCGTTGCGCTACACTCT | CTGAAGTCGTAGCAGTGTTGCT |
| ORF35 | GTAGTCAGAGTCTGAACCCCGT | GGCCATGGGCTGAATATCTT |
| ORF40 | CGTATGATATCCCTATCGCAGG | CACTTGCTGTCCTCATTGATCC |
| ORF44 | CGGAAGGGTGCACTGATATG | TACGCTCCTGACTTTGGCA |
| ORF54 | GTGGAACGCAGACACATCCT | AAAATGCACTCGAGCCTCC |
| ORF56 | AGTTCGTCCTGACCAGCTCA | GCGAAAACAGTCTAGACAGGTG |
| ORF57 | ATGACGACGTCAGACAGGGT | GGGCTCTCACCGTCAATTAT |
| ORF63 | CTGGAATCATCGGCCTGTT | TGTCAGTATCCACATTGGGG |
| ORF64 | TACCGCACCATTGTGTTCG | GATTTCGGTGATGGAGCATG |
| ORF69 | AGTGGACTTTCTCCGTGAGATG | TGCGACGGTAGACAGGTCTA |
| ORFK8.1 | CTGTTGAAGATACGTCTGCCTC | AAAGTCACGTGGGAGGTCAC |
| ORFK14 | GATGGGTACTGACATCCGCT | AACACATGGCCTGCTTGCT |
| DNMT1 | TCCGATGGAGAGGCTAAGCG | GATGGTGGTTTGCCTGGTGC |
| DNMT3A | GGAGCCGCGTGGCAAGGAGG | CACCGCAGGGTCCTTTGGCG |
| DNMT3B | CGACTCGCCCCCAATCCTGG | CGCCATCGCCTGTCAAGTCC |
