# Supplementary material for: Metabolic reprogramming of Kaposi’s sarcoma associated herpes virus infected B-cells in hypoxia
Source: PLoS Pathog. 2018 May 10;14(5):e1007062. doi: 10.1371/journal.ppat.1007062 (PMC5963815; doi:10.1371/journal.ppat.1007062)
Supplement: S4 Table — (DOCX) [file ppat.1007062.s008.docx]

**S4 Table:** Primers used to amplify different HREs containing promoter regions.

| HRE | Forward Primer (5’-3’) | Reverse Primer (5’-3’) |
| --- | --- | --- |
| C1 | ATATCTCGAGTAAGTCAGCCGGACCAAGCTGC | ATATAAGCTTAGTTTCATTCCAGGATTCATCA |
| C2 | ATATCTCGAGTAAGTCAGCCGGACCAAGCTGC | ATATAAGCTTGCCTGGGGCACCAATCAGAAAG |
| C3 | ATATCTCGAGTAAGTCAGCCGGACCAAGCTGC | ATATAAGCTTGGCGACGTTTACGGGACCGGGGA |
| C4 | ATATCTCGAGTAAGTCAGCCGGACCAAGCTGC | ATATAAGCTTTGTGGCGGTACTGGGTCTGAAC |
| C5 | ATATCTCGAGTAAGTCAGCCGGACCAAGCTGC | ATATAAGCTTGTAGGGGGAGATGTCACTTCCA |
| C6 | ATATCTCGAGTAAGTCAGCCGGACCAAGCTGC | ATATAAGCTTCATCACTGTCAGTGAACTGCTC |
| C7 | ATATCTCGAGGTGTCTAGCTGGTCCCGTGGTC | ATATAAGCTTAGTTTCATTCCAGGATTCATCA |
| C8 | ATATCTCGAGCCGTCAGGGGAGTGACAAGCTA | ATATAAGCTTAGTTTCATTCCAGGATTCATCA |
| C9 | ATATCTCGAGCACCTGGCCCAAACGGAGGATC | ATATAAGCTTAGTTTCATTCCAGGATTCATCA |
| C10 | ATATCTCGAGGGGTGTGCATTTATTAGACG | ATATAAGCTTAGTTTCATTCCAGGATTCATCA |
