# Supplementary material for: Metabolic reprogramming of Kaposi’s sarcoma associated herpes virus infected B-cells in hypoxia
Source: PLoS Pathog. 2018 May 10;14(5):e1007062. doi: 10.1371/journal.ppat.1007062 (PMC5963815; doi:10.1371/journal.ppat.1007062)
Supplement: S5 Table — (DOCX) [file ppat.1007062.s009.docx]

**S5 Table:** Real time PCR primers used to validate RNA sequencing fold change results:

| Gene | Forward Primer | Reverse Primer |
| --- | --- | --- |
| ALDOA | CCAAGGGCGGTGTTGTGGGC | CTTCTTGTACTGGGCACAGC |
| ALDOB | CAAGGTGGAAAACACTGAAG | GGTCTCGTGGAAAAGGATCA |
| ENO1 | GCCATGCAGGAGTTCATGAT | CCATATTTCTCCTTGATGAC |
| ENO2 | GGAGCTGAGGGATGGAGACA | CCACAGAGAGACCTGAGCTG |
| HK2 | CAAAGTGACAGTGGGTGTGG | GCCAGGTCCTTCACTGTCTC |
| IDH3B | GGAGAAGCTGGAGCAGGTGC | GCATATCATAGGAGGCTAGC |
| MDH1 | GGATGTGGCCATTCTTGTGG | ATCTAAGGCTGCACCCTGGG |
| PCK1 | GGAGTTTGTCAAATGCCTCC | GGTGGGCGATGAGCGTCAGC |
| PDK3 | CCTTTCATTAGCACTAACAT | GTCACCCCCAAACAGAAGTG |
| PDP2 | CACCCTAAACAGTTCCCCAT | TCTGCTCAGGGCTGAGTTGC |
| PFKL | GTCATCGATGCCATCACCAC | CCTGAGGCCAGTGCAGATAC |
| PGK1 | CCTGCTGGAGAACCTCCGCT | AGTGAAGCTCGGAAAGCTTC |
| PRPS1 | CAATCTGCCATGCAGCTGAC | ACTACTGCCTCAAAGCATGC |
| PRPS2 | CTCATCATGATCAATGCCTG | CACGACTCTCTCCTACCTTG |
| RPE | CCAGGAACCTCAGTTGAGTA | TCTTCCATGAATTTCTGCCC |
| TPI1 | TGGGGCACTCAGAGAGAAGG | GCTTCTCCCCAATGCAGGCG |
| BPGM | CTGGAAGAGCTAGGCCAGGA | GCAGATGGCTTTGAATCATG |
| DLST | GGTTGTCATTAACAACAGTG | GTGACAGATTCTGCAAACGC |
| GALM | CCCGGAGAGTTAAAAGTCTG | AAGAATGGTTGGTCAGGTTG |
| GSK3A | GGCTTACACGGACATCAAAG | GGAGAACCTTCTTGATGGCG |
| GSK3B | TCCAGACAGGCCACAAGAAG | TCCTGAATCACAAAGTTTGG |
| IDH1 | CACCAAATGGCACCATACGA | GGTTTTACCCATCCACTCAC |
| PDPR | CTGTGGAGGTGGAATCACGG | AGAGCCAGCAGCCAGCCTGC |
| PGM3 | GGATTATTAGCTGTCCTGAG | GGATCAACCAATTTTACACC |
| PHKG1 | GCTGGGACTCTCCCAAGTCC | CCTTCTCACTCAAGGTGACC |
| PHKG2 | GACAGCTGAGCGGCTGAGTC | GGTGATGATGTGGGGGTGGC |
| PYGM | CCGTGCGCACGAACTTCGAT | CGATGAGGATCCTGGTGGACC |
| PGK2 | CCTGCTGGAGAACCTGCGCT | AGTGATGCTCGGAAGGCTTC |
| PHKB | CTTGAGAGATGGGTATAGAAC | GGAAATTCACATTCAATGCC |
| IDH3B | GGAGAAGCTGGAGCAGGTGC | GCATATCATAGGAGGCTAGC |
| TALDO1 | GATGGTGGCCAGAGCCAGGC | AGCCTGAATTCCTTCCCAGG |
| ACO2 | GCTTCTGGAAGCCTGGATCT | CACCATTGGGGGTGTGGGAG |
| AGL | GTACATGTTGGATGCTGCTA | GTAACAAAGACATTGTCCAG |
| GBE1 | GCATTCAGTTGATGGCAATC | TGTAGCTCTTCAGGTGTTCC |
| IDH2 | GTGGAAGAGTTCAAGCTGAAG | GGATGTTTTTGCAGATGATG |
| OGDH | GAAGGCTGCGAGGTACTGATC | TCAGCCGCCCTCTGTGTGGC |
| PCK2 | GGACACGGTACCACTCCCGC | ATGCAGCCTGGAAACCTCTC |
| PDHB | CCTATAGTCTTCAGGGGGCC | GGACTGACCACCTTTAAGCC |
| PGK2 | CCTGCTGGAGAACCTGCGCT | AGTGATGCTCGGAAGGCTTC |
| PGM1 | CCCAATGGAGATTTTGGAATC | GGGCAAACTGCATATTCTTC |
| SDHA | CGCACTGTGCATAGAGGACG | TGTGGGCAGACGTGCAGCTG |
| SUCLG2 | CATTTTTAAGGAGCAAATTG | GGCTTTTCAAAGGCCCAACG |
| TKT | CACCGACGTGGCCACTGGCT | CAAGCAATAGACTCGGTAGC |
| ACLY | GGAGTTTGTGAACAAGATGA | ATCTGCACTCGCATGTCTGG |
| ACO1 | CACTGACATCGTGCTCACCA | GGTCAGCAATGGACAACTGG |
| ALDOC | GATGGTGCTGACTTTGCCAAG | AGATACTGGCATAACGGGCC |
| CS | GGCATGAGAGGCATGAAGGGA | TCCCTTCATGCCTCTCATGCC |
| DLAT | GAGGAGTGTTATATGGCAAAG | AGGCCTCAATATCCTCAGGC |
| DLD | CTACGAAAGCTGATGGCGGC | CTATTGTATCTTCATCTATCG |
| ENO3 | GGAACTAAGAGACGGAGACA | CCCTTCTCAGCTGCTCCCGC |
| FH | CAGACCGTGAGATCTACGAT | TTACTTCAGCGGCCGCTCGC |
| G6PC3 | CAGCCCAGGTTCACCAGTTC | AGACAGGGCCGTCATTATGG |
| GPI | CATCAACTGCTTTGGGTGTG | TGTATTTCCCATTGGACTCC |
| GYS1 | GTGGTTGGCAGGCGTTGGAC | CACCGGCACACAGGTAGCGC |
| H6PD | CCCTCTCCTGCCCCAAGGAC | GATAGTCCTCGGCCGTCTTC |
| IDH3A | GCTTGAAAGGCCCTTTGAAG | ATAGAGACACATGGTCGGAC |
| IDH3G | CATCGAAACCAACCATAACC | CACGCCTGGAAGGCTCTTAC |
| MDH1B | GCATAACTCTATTTGACAAC | TCGTGCAGATGGAGACACTG |
| MDH2 | GCCAATCCGGGTTTGGATCC | AAAGTCCACCTTGGGGGTGC |
| PC | CCAAGCCCAGAAGTGGTCCG | CGTGATGGGGGCATCTGTGC |
| PDHA1 | GTGATGGTCAGGAAGCTTGC | GCCCCGGGTGAAAGTAAAGC |
| PDK1 | GGATCAGAAACCGACACAAT | ACATTCTGGCTGGTGACAGG |
| PDK3 | CCTTTCATTAGCACTAACAT | GTCACCCCCAAACAGAAGTG |
| PGLS | GCCTGTGGAGGAGGCGGCTG | CATCGGGGCCCACCCCCAGG |
| PGM2 | CATCTCACAATCCAAAGCAG | TCTAGATTTTCTTCAATAGC |
| PHKA1 | CAGGGTCGTTATGGTTGCTG | AATAGCTTCAGCTCAGCTGG |
| RPIA | CAGTATGGCTTGACCCTCAG | CTTGATGAGATTGAGATCAG |
| SDHC | CACCGTGGCACTGGTATTGC | AACCAGGACAACCACTCCAG |
| SUCLA2 | GTTCCCAAAGGATATGTGGC | CTCTACCACCAGCTAAAACC |
